# Using large models and time series forecasting for market reaction analysis

**DOI:** 10.1371/journal.pone.0356139

**Published:** 2026-09-30

**Authors:** Hong He, Xinhai Li

**Affiliations:** 1 School of Economics and Management, Harbin Petroleum Institute, Harbin, Heilongjiang, China; 2 Accounting college, Harbin University of Commerce, Harbin, Heilongjiang, China; Philadelphia University, JORDAN

## Abstract

Forecasting financial market movements requires integrating numerical time-series data with semantic information from financial narratives that shape investor behavior. While deep learning has advanced time-series prediction, existing methods often exploit trivial price autocorrelation rather than capturing genuine predictive signals from multimodal sources. We propose the Semantic-Time Fusion Architecture (STFA), which integrates structured market data with GPT-4-supervised FinBERT semantic representations of financial news through three key innovations: delay-aware temporal alignment between text publication and market reaction, semantic-guided attention mechanisms emphasizing news-relevant historical patterns, and multi-scale temporal fusion with multi-objective training to balance accuracy and interpretability. We evaluate our model on a large-scale financial news and stock price dataset using GPT-4 sentiment annotation across stratified high-liquidity stocks with strictly chronological train-validation-test splits (2013–2020 for training, 2021 for validation, 2022–2023 for testing). Sentiment annotations were generated independently for each article without using any future market information, ensuring strict temporal separation and preventing information leakage. Experiments demonstrate that STFA significantly outperforms traditional time-series models, multimodal baselines, and random walk benchmarks on return prediction, where price autocorrelation vanishes, rather than relying on price-level forecasting. Statistical validation with bootstrap confidence intervals, effect size analysis, and Bonferroni-corrected significance tests confirms robust improvements across independent runs. Ablation studies reveal that GPT-4-supervised FinBERT semantic representations provide superior signals compared with lexicon-based sentiment, with each architectural component contributing significantly to performance. This work demonstrates how temporally aligned semantic understanding enhances both predictive accuracy and model interpretability in financial forecasting.

## Introduction

Accurately forecasting stock price movements is critical to decision-making in financial markets, underpinning everything from individual investment strategies to large-scale institutional risk management [1–3]. Reliable forecasts guide capital allocation and shape how market participants respond to uncertainty, volatility, and emerging information. Traditional forecasting models, including recurrent neural networks (RNNs), long short-term memory networks (LSTMs), and Transformer-based architectures, have largely relied on structured numerical data such as historical prices, trading volumes, and technical indicators [4–7]. While these models effectively identify recurring temporal patterns, they often overlook a crucial dimension of financial decision-making: the influence of textual information.

Market dynamics are not solely dictated by numerical trends. Sudden price swings often originate from qualitative factors such as corporate disclosures, policy announcements, or macroeconomic reports that contain subtle cues capable of shifting investor sentiment [8]. These textual signals may not immediately affect technical indicators, yet they can trigger sharp market reactions. For instance, news of regulatory delays or ambiguous forward guidance may appear neutral in tone but still introduce uncertainty leading to significant valuation changes. Such reactions expose a fundamental limitation of purely numerical models: their inability to interpret the semantics embedded in financial language.

To address this gap, prior work has explored incorporating sentiment information derived from financial news. Lexicon-based tools such as TextBlob and VADER assign polarity scores to text, augmenting numerical features with simple sentiment indicators. However, these approaches often fail to capture domain-specific nuances or contextual complexity. A sentence describing missed revenue expectations alongside record profits may be classified as neutral despite implying investor disappointment, introducing noise that can degrade forecasting accuracy.

The emergence of large language models (LLMs), such as ChatGPT, presents new opportunities for understanding financial narratives. These models can extract rich semantic information from unstructured text, including sentiment, event type, causality, and temporal implications [9,10]. Unlike traditional sentiment tools, LLMs can reason over language, interpret implicit cues, and provide structured insights aligned with market logic. In this study, GPT-4 is used to provide high-quality training-period sentiment supervision, while a GPT-4-supervised fine-tuned FinBERT encoder generates the semantic representations used by the forecasting model. Effectively integrating such semantic representations into time-series forecasting presents several challenges: aligning textual and numerical data across time, handling delayed market responses, identifying relevant historical context, capturing reactions across multiple temporal horizons, and ensuring model interpretability.

To address these challenges, we propose the Semantic-Time Fusion Architecture (STFA), a framework designed to integrate structured financial data with GPT-4-supervised financial semantic representations extracted from news. STFA incorporates four main components. First, a financial semantic encoding module constructs structured semantic vectors from financial text. Second, a delay-aware temporal alignment module accounts for temporal deferral between textual signals and market reactions. Third, a semantic-guided attention mechanism identifies which historical time-series patterns are most relevant given the current semantic signal. Fourth, a multi-scale temporal fusion module captures market responses across different time horizons. These components are trained with a multi-objective strategy that jointly optimizes predictive accuracy, semantic consistency, and interpretability.

We evaluate STFA using the Financial News and Stock Price Integration Dataset (FNSPID) [11], which contains over 15 million financial articles covering thousands of companies spanning more than two decades. We construct a focused experimental corpus by selecting 50 high-capitalization S&P 500 stocks with dense news coverage from 2013 to 2023, with training period (2013–2020), validation period (2021), and test period (2022–2023). We apply GPT-4-based sentiment annotation using the gpt-4–0613 model with temperature set to 0.0 to training-period articles used for supervision, and then use the fine-tuned FinBERT encoder to generate semantic representations consistently across training, validation, and test periods. Critically, all sentiment annotations and semantic representations are generated independently for each article based solely on its content, without using any future market information or outcomes, ensuring strict temporal separation and preventing information leakage.

Experimental results demonstrate that STFA significantly outperforms conventional time-series models and multimodal baselines across multiple evaluation metrics. Ablation studies reveal the contribution of each architectural component, confirming the importance of semantic encoding, delay modeling, semantic-guided attention, and multi-scale temporal fusion. This work contributes a framework that bridges numerical analysis and narrative reasoning in financial forecasting. By integrating structured market data with contextual semantic representations, STFA improves both prediction accuracy and interpretability. This approach demonstrates how intelligent accounting and financial AI systems can learn not only from numerical patterns but also from the contextual narratives that drive market behavior.

## Related work

The integration of textual information with financial time-series forecasting has been explored across multiple disciplines including finance, natural language processing, and machine learning. We review relevant work in three main areas: time-series forecasting models, textual sentiment and event modeling, and multi-modal fusion techniques.

### Time-series forecasting models

Classical approaches to financial forecasting primarily rely on numerical data using statistical methods such as ARIMA, GARCH, and Kalman filters, building upon foundational theories of market efficiency and asset pricing [12,13]. These methods capture linear trends and volatility but often lack flexibility when handling non-stationary behaviors or long-term dependencies. Deep learning techniques, including Recurrent Neural Networks (RNN), Long Short-Term Memory networks (LSTM), and Gated Recurrent Units (GRU), have become widely used for time-series prediction due to their capacity to model complex sequential patterns. More recently, attention-based architectures such as Transformer and its variants have demonstrated strong performance in capturing global temporal dependencies, particularly in long-horizon forecasting scenarios [4,5].

Hanssens et al. [14] provided comprehensive treatment of market response modeling using econometric and time series analysis, establishing foundations for understanding how structured quantitative models can forecast consumer behavior and marketing interventions. Hanssens [15] applied ARMA models to analyze competitive reactions and primary demand within markets, demonstrating that time series models can capture nuanced interactions between competing firms and dynamic feedback loops in market behavior. Dekimpe and Hanssens [16] reviewed time series modeling in marketing, identifying trends and challenges in adapting these models to changing market conditions. They emphasized the importance of structural flexibility and highlighted limitations of traditional models under shifting consumer patterns. Zhao et al. [17] introduced a Time Series Relational Model (TSRM) designed to account for linkage effects across stocks, integrating temporal dependencies and cross-stock relationships. Tsai et al. [18] proposed a hybrid forecasting model combining statistical and machine learning methods to predict leading industry stock prices. Montgomery et al. [19] provided practical and theoretical guidance on time series analysis and forecasting, including applications to financial data.

Despite these advances, these methods remain fundamentally limited by their reliance on structured numerical data alone, without incorporating information from textual sources.

### Textual sentiment and event modeling

Recognizing the potential impact of unstructured financial narratives, numerous studies have explored augmenting forecasts with text-derived sentiment. Early efforts employed lexicon-based sentiment tools such as Loughran-McDonald, VADER, and TextBlob to compute polarity scores from financial news, earnings calls, or social media posts. While accessible, these methods often oversimplify sentiment and fail to capture context, domain-specific expressions, or nuanced language. Other work has employed machine learning classifiers trained on labeled corpora, such as FinBERT, which provide more accurate sentiment detection and event classification. Event-based models incorporating named entity recognition or trigger-based event extraction aim to identify impactful corporate actions including mergers, lawsuits, and earnings surprises.

Kearney and Liu [20] conducted a comprehensive survey on textual sentiment analysis in finance, categorizing models based on their application to individuals, firms, and markets. They discussed how market responses can be shaped by sentiment-linked events and examined methodological trends across lexicon-based, statistical, and machine learning approaches. Daniel et al. [21] developed methods to measure event popularity using sentiment analysis of social media content. Celuch [22] applied topic modeling and sentiment analysis to online reviews, focusing on consumer reactions to event ticket purchases, demonstrating how sentiment tools can extract meaningful insights from user-generated content. Todd [23] explored investor behavior in reaction to sentiment-laden events, finding that markets often exhibit asymmetric responses to positive versus negative events. Jiang et al. [24] proposed a stakeholder-based event analysis framework integrating social media sentiment with market responses. Makrehchi et al. [25] introduced an event-based sentiment analysis model for stock prediction by automatically detecting significant market events. Valle-Cruz et al. [26] examined how social media sentiment influenced stock decisions during health crises.

However, many of these approaches operate independently of forecasting models and are not directly optimized for downstream financial prediction tasks.

### Multi-modal fusion techniques

Recent research has explored fusion methods that integrate numerical and textual data. Some approaches concatenate textual features with numerical indicators as input, while others implement dual-stream architectures that separately encode news and price data before merging them via attention mechanisms. Examples include hierarchical attention networks, cross-modal transformers, and co-attention frameworks.

Qin [27] proposed MSMF, a Multi-Scale Multi-Modal Fusion framework for stock market prediction, incorporating diverse financial signals across multiple temporal and data modalities. Yadav [28] presented a Transformer-based architecture integrating financial news sentiment and social media signals with numerical stock indicators. Zhu et al. [29] surveyed multimodal sentiment analysis, focusing on integration techniques for combining textual, visual, and acoustic modalities. AI et al. [30] introduced MMF-Trans, a multi-modal Transformer model for the Chinese stock market, fusing macroeconomic news with firm-level data. Li et al. [31] developed M2TFM, a Multi-Modal Transformer Fusion Model for forecasting new product sales, demonstrating methodology transferable to financial contexts.

Recent advances have introduced more sophisticated fusion mechanisms specifically designed for financial markets. Zong et al. [32] proposed a gated cross-attention mechanism for multimodal stable fusion in stock movement prediction, demonstrating improved stability when integrating heterogeneous data sources. Wang [33] developed an improved feedforward neural network architecture with multimodal fusion capabilities, addressing challenges in capturing complex nonlinear relationships between textual sentiment and price dynamics. Wang et al. [34] focused on multimodal market information fusion for pharmaceutical sector stock prediction, highlighting sector-specific considerations in corpus design and feature engineering. Taylor and Ng [35] explored natural language processing techniques combined with multimodal architectures for stock price prediction, emphasizing the importance of linguistic context in financial narratives. Liu et al. [36] introduced multiscale dynamic graph convolution networks that model stock interdependencies alongside multimodal features, capturing both semantic signals and relational market structure.

Despite these architectural innovations, several critical limitations persist across existing multimodal forecasting frameworks. Many models overlook temporal misalignment between news publication and market reaction, treating semantic signals as instantaneous rather than accounting for propagation delays that characterize real-world information diffusion. Few approaches explicitly model the lag between textual information release and observable price impact, potentially missing critical dynamics in how narratives gradually influence investor behavior and trading decisions. Furthermore, most existing frameworks lack interpretable mechanisms to explain which specific textual elements or temporal patterns contribute most significantly to predictions, limiting their utility in practical decision-making contexts where transparency and regulatory compliance are essential.

## Methodology

### Model overview

The Semantic-Time Fusion Architecture (STFA) integrates financial textual information with structured market time-series data for market reaction analysis, as illustrated in Fig 1. The framework first converts financial news into structured semantic representations. In our implementation, GPT-4 is used to generate training-period sentiment labels, and a GPT-4-supervised fine-tuned FinBERT encoder is used to produce contextual article embeddings for model input. This separation allows the model to benefit from high-quality GPT-4 supervision while using a fixed and reproducible encoder for large-scale semantic feature generation.

**Fig 1:**
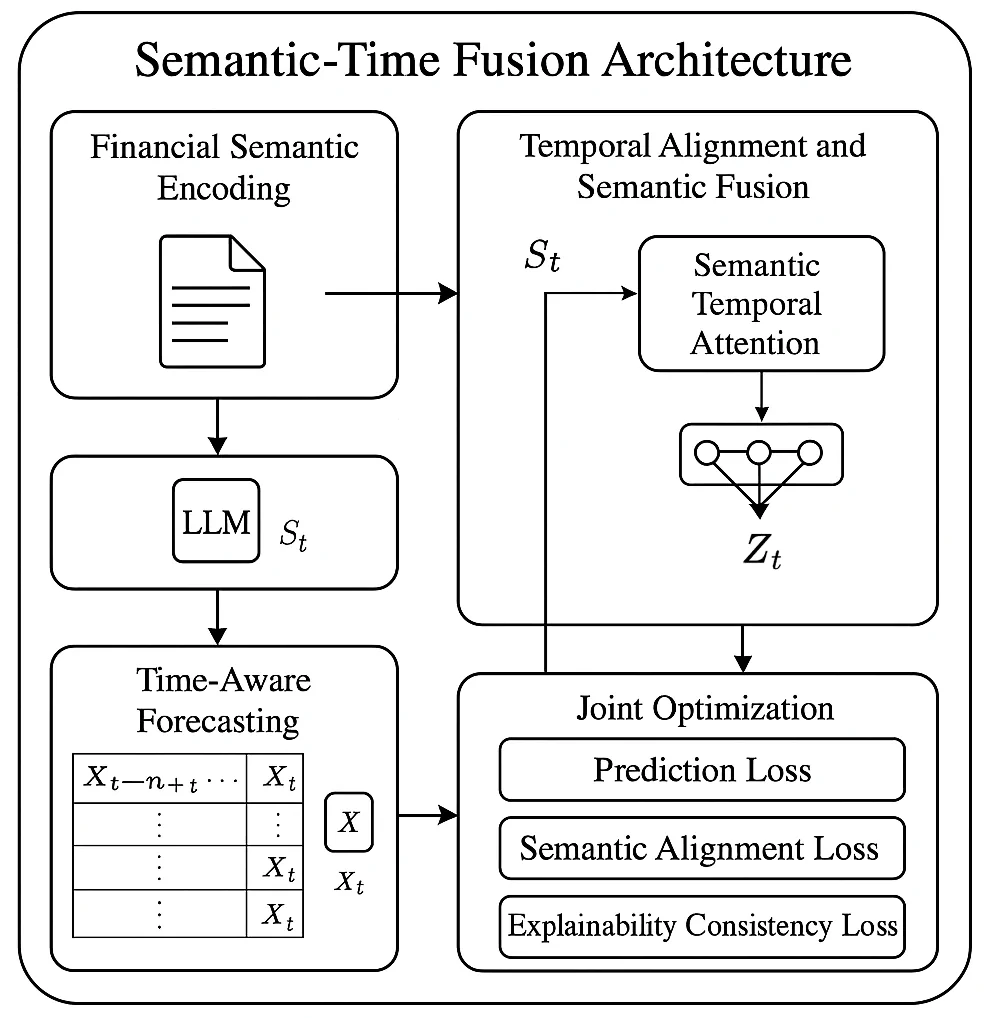
Overview of Semantic-Time Fusion Architecture. Financial text is encoded into structured semantic representations, aligned with historical time-series data through delay-aware fusion and semantic-guided attention, processed by multi-scale temporal fusion, and passed to a forecasting module trained with a multi-objective loss.

STFA contains four main components. First, the financial semantic encoding module aggregates article-level semantic embeddings and projects them into a structured semantic representation. Second, the temporal alignment and semantic fusion module estimates a learned delay factor and applies semantic-guided attention over historical market observations. Third, the multi-scale temporal fusion module captures market reactions across multiple temporal horizons. Fourth, the forecasting module combines self-attention and multi-scale temporal representations to predict future market outcomes. The model is trained using a multi-objective loss that balances forecasting accuracy, semantic consistency, and interpretability.

Throughout this section, we use distinct notation for different feature spaces to avoid dimensional ambiguity. The raw FinBERT article embedding dimension is denoted by *d*_BERT_ = 768. The structured semantic dimension used inside STFA is denoted by $d_{s}=128$. The model hidden dimension used by attention, temporal fusion, and forecasting layers is denoted by $d_{h}=128$. The raw numerical market feature dimension is denoted by *p*.

### Financial semantic encoding

The Financial Semantic Encoding module transforms unstructured financial language into structured semantic vectors that can be integrated with quantitative market data, as shown in Fig 2. Let $T_{t}=\{\tau_{t}^{\left(1\right)},\tau_{t}^{\left(2\right)},...,\tau_{t}^{\left(N_{t}\right)}\}$ denote the set of financial documents associated with time step *t*, where each document may be a news article, earnings-related repor*t*, or regulatory filing linked to a stock.

**Fig 2:**
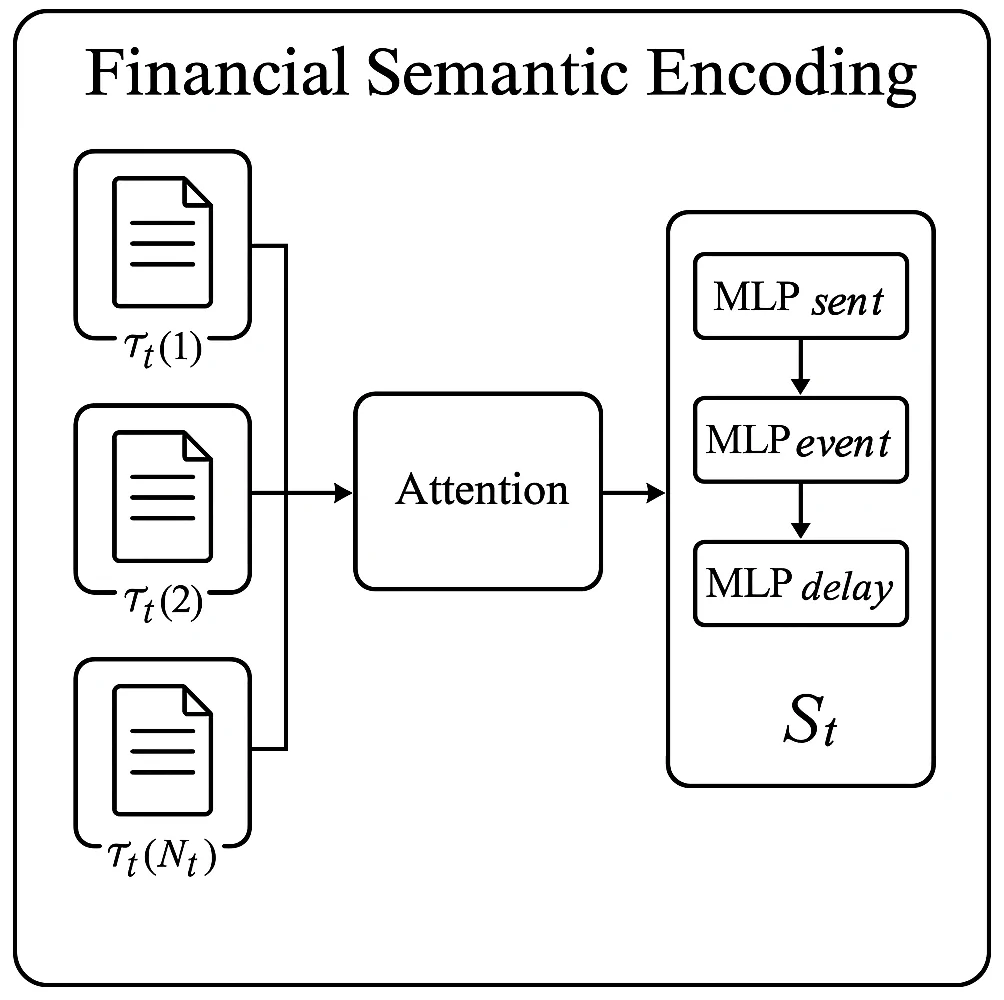
Financial Semantic Encoding. Multiple financial documents within a time window are encoded by a GPT-4-supervised fine-tuned FinBERT encoder, aggregated through length-weighted averaging, and projected into structured semantic subspaces representing sentiment, event type, and temporal delay.

Each document $\tau_{t}^{\left(i\right)}$ is processed by a semantic encoder *F*_sem_. In our implementation, *F*_sem_ is the fine-tuned FinBERT encoder supervised by GPT-4-generated training-period sentiment labels:


$$\begin{array}{c} h_{t}^{\left(i\right)}=F_{\text{sem}}(\tau_{t}^{\left(i\right)})\in {\mathbb{R}}^{d_{\text{BERT}}}, \end{array}$$ (1)

where *d*_BERT_ = 768 denotes the dimensionality of the FinBERT CLS-token representation. The symbol *d*_BERT_ is used only for the original semantic encoder output dimension.

When multiple documents are associated with the same stock-day window, we aggregate their embeddings using length-weighted averaging:


$$\begin{array}{cc} w_{t}^{\left(i\right)} & =\frac{\text{WordCount}(\tau_{t}^{\left(i\right)})}{\sum_{j=1}^{N_{t}}\text{WordCount}(\tau_{t}^{\left(j\right)})},\\ S_{t} & =\sum_{i=1}^{N_{t}}w_{t}^{\left(i\right)}h_{t}^{\left(i\right)},\;S_{t}\in {\mathbb{R}}^{d_{\text{BERT}}}. \end{array}$$ (2)

Here, $w_{t}^{\left(i\right)}$ is the normalized word count of document *i*. This aggregation gives greater weight to longer documents, which generally contain more detailed financial information, while avoiding additional trainable aggregation parameters.

The aggregated embedding $S_{t}$ is then projected into three semantic subspaces:


$$\begin{array}{c} S_{t}^{\text{sent}}={\text{MLP}}_{\text{sent}}(S_{t}),\;S_{t}^{\text{event}}={\text{MLP}}_{\text{event}}(S_{t}),\;S_{t}^{\text{delay}}={\text{MLP}}_{\text{delay}}(S_{t}). \end{array}$$ (3)

These subspaces represent sentiment polarity, event type, and temporal deferral information, respectively. The sentiment subspace captures the market tone of the text, the event subspace captures the type of corporate or macroeconomic information, and the delay subspace captures text-derived information related to the expected timing of market impact.

The three subspace outputs are concatenated into an intermediate structured semantic vector:


$$\begin{array}{c} {\bar{S}}_{t}=[S_{t}^{\text{sent}};S_{t}^{\text{event}};S_{t}^{\text{delay}}]. \end{array}$$ (4)

In the released reproducibility features, this intermediate vector has dimension 129, consisting of 64 sentiment dimensions, 64 event dimensions, and one delay-related scalar. Before entering the fusion module, this intermediate vector is projected into the fixed structured semantic dimension used by STFA:


$$\begin{array}{c} {\tilde{S}}_{t}=W_{c}{\bar{S}}_{t}+b_{c},\;{\tilde{S}}_{t}\in {\mathbb{R}}^{d_{s}}. \end{array}$$ (5)

In our implementation, $d_{s}=128$. This structured semantic dimension is distinct from *d*_BERT_ = 768, the original FinBERT embedding dimension, and from $d_{h}=128$, the model hidden dimension used in the fusion and forecasting layers.

### Temporal alignment and semantic fusion

After obtaining the structured semantic vector ${\tilde{S}}_{t}$, STFA aligns it with historical market observations and constructs a fused semantic-time representation. Let $X_{t-n:t}=\{x_{t-n},...,x_{t}\}$ denote the historical time-series window, where each $x_{i}\in {\mathbb{R}}^{p}$ contains OHLCV variables and technical indicators. The structured semantic vector is ${\tilde{S}}_{t}\in {\mathbb{R}}^{d_{s}}$, where $d_{s}=128$.

To model delayed market reactions to textual information, we estimate a scalar delay factor from the structured semantic vector:


$$\begin{array}{c} D_{t}=f_{\text{delay}}({\tilde{S}}_{t}),\;D_{t}\in \mathbb{R}, \end{array}$$ (6)

where *f*_delay_ is a learnable MLP. The delay-related component inside the intermediate semantic input ${\bar{S}}_{t}$ is a text-derived feature, whereas $D_{t}$ is a learned temporal-alignment estimate used by the fusion module. Thus, $D_{t}$ is not a duplicate stored feature but a trainable transformation of the structured semantic representation. The delay estimate allows the model to represent different lag structures for different types of financial information.

Next, STFA applies semantic-guided temporal attention. Because raw numerical observations and semantic vectors have different dimensions, both modalities are first projected into a shared hidden space:


$$\begin{array}{c} u_{i}=W_{x}x_{i}+b_{x},\;u_{i}\in {\mathbb{R}}^{d_{h}}, \end{array}$$ (7)

$$\begin{array}{c} v_{t}=W_{s}{\tilde{S}}_{t}+b_{s},\;v_{t}\in {\mathbb{R}}^{d_{h}}, \end{array}$$ (8)

where $W_{x}\in {\mathbb{R}}^{d_{h}\times p}$, $W_{s}\in {\mathbb{R}}^{d_{h}\times d_{s}}$, and $d_{h}=128$ in our implementation.

The compatibility between each historical market state and the current semantic signal is computed in the shared hidden space using scaled dot-product attention:


$$\begin{array}{c} e_{i}=\frac{\left(W_{q}u_{i}{)}^{⊤}(W_{k}v_{t}\right)}{\sqrt{d_{h}}},\;i\in \{t-n,...,t\}, \end{array}$$ (9)

$$\begin{array}{c} \alpha_{i}=\frac{\exp(e_{i})}{\sum_{j=t-n}^{t}\exp(e_{j})}. \end{array}$$ (10)

The attention weight $\alpha_{i}$ is defined over historical time steps, indicating which parts of the numerical history are most compatible with the current semantic signal.

The attention context is computed as a weighted sum of projected value vectors:


$$\begin{array}{c} A_{t}=\sum_{i=t-n}^{t}\alpha_{i}W_{v}u_{i},\;A_{t}\in {\mathbb{R}}^{d_{h}}, \end{array}$$ (11)

where $W_{q},W_{k},W_{v}\in {\mathbb{R}}^{d_{h}\times d_{h}}$ are learnable projection matrices. Since the value vectors are in the model hidden space, the resulting context vector $A_{t}$ is also in ${\mathbb{R}}^{d_{h}}$.

Finally, the fused representation is constructed by concatenating the current market observation, the structured semantic vector, the scalar delay estimate, and the semantic-guided attention context:


$$\begin{array}{c} Z_{t}=[x_{t};{\tilde{S}}_{t};D_{t};A_{t}]. \end{array}$$ (12)

Because $x_{t}\in {\mathbb{R}}^{p}$, ${\tilde{S}}_{t}\in {\mathbb{R}}^{d_{s}}$, $D_{t}\in \mathbb{R}$, and $A_{t}\in {\mathbb{R}}^{d_{h}}$, the fused vector has dimension


$$\begin{array}{c} Z_{t}\in {\mathbb{R}}^{p+d_{s}+1+d_{h}}. \end{array}$$ (13)

This explicit dimensional definition distinguishes the raw numerical dimension, structured semantic dimension, scalar delay term, and model hidden dimension.

### Multi-scale temporal fusion

Market reactions may occur over different temporal horizons. Some news events affect prices within one or two trading days, while earnings guidance, regulatory investigations, and macroeconomic announcements may produce slower adjustments. To capture these horizon-dependent patterns, STFA includes a multi-scale temporal fusion module.

Let $T_{\text{fusion}}=\{Z_{t-k},...,Z_{t}\}$ denote the sequence of fused semantic-time representations over a lookback window, where each $Z_{i}\in {\mathbb{R}}^{p+d_{s}+1+d_{h}}$. Each fused vector is first projected into the model hidden space:


$$\begin{array}{c} G_{i}=W_{z}Z_{i}+b_{z},\;G_{i}\in {\mathbb{R}}^{d_{h}}. \end{array}$$ (14)

The projected sequence is processed by multiple temporal branches with different receptive fields:


$$\begin{array}{c} M_{t}^{\left(r\right)}=\phi_{r}(G_{t-r+1:t}),\;M_{t}^{\left(r\right)}\in {\mathbb{R}}^{d_{h}},\;r\in ℛ, \end{array}$$ (15)

where ℛ={2,5,10,20} denotes temporal scales from short to long horizons. The function $\phi_{r}(\cdot )$ is a scale-specific temporal encoder implemented using one-dimensional convolution followed by GELU activation and layer normalization. Each branch outputs a vector in the shared hidden dimension $d_{h}$, allowing the scale-attention mechanism to combine branches by weighted summation.

The outputs of the temporal branches are combined through learned scale attention:


$$\begin{array}{c} \beta_{r}=\frac{\exp(w_{m}^{⊤}M_{t}^{\left(r\right)})}{\sum_{r^{\prime }\in ℛ}\exp(w_{m}^{⊤}M_{t}^{\left(r^{\prime }\right)})}, \end{array}$$ (16)

$$\begin{array}{c} M_{t}=\sum_{r\in ℛ}\beta_{r}M_{t}^{\left(r\right)},\;M_{t}\in {\mathbb{R}}^{d_{h}}. \end{array}$$ (17)

Here, $\beta_{r}$ represents the learned contribution of scale *r*, and $M_{t}$ is the final multi-scale temporal representation. This module corresponds to the “+ Multi-scale Fusion” component evaluated in the ablation study.

### Time-aware forecasting

The forecasting module predicts future market outcomes from both the self-attention representation of the fused sequence and the multi-scale temporal representation. Given $T_{\text{fusion}}=\{Z_{t-k},...,Z_{t}\}$, the model first embeds the sequence and applies multi-head self-attention:


$$\begin{array}{cc} H^{\left(0\right)} & =\text{Embed}(T_{\text{fusion}}),\\ H^{\left(l\right)} & =\text{MHSA}(H^{\left(l-1\right)}),\;l=1,...,L. \end{array}$$ (18)

The embedding layer maps each fused vector $Z_{i}$ into the model hidden space, so *H*^(0)^ and all subsequent self-attention states are represented in ${\mathbb{R}}^{d_{h}}$. The final hidden state corresponding to the current time step is denoted as $H_{t}^{\left(L\right)}\in {\mathbb{R}}^{d_{h}}$.

The multi-scale temporal fusion module produces $M_{t}\in {\mathbb{R}}^{d_{h}}$. We concatenate the self-attention output and the multi-scale representation:


$$\begin{array}{c} O_{t}=[H_{t}^{\left(L\right)};M_{t}],\;O_{t}\in {\mathbb{R}}^{2d_{h}}. \end{array}$$ (19)

The final prediction is generated by a task-specific output decoder:


$$\begin{array}{c} {\hat{Y}}_{t+1:t+H}={\text{MLP}}_{\text{out}}(O_{t}), \end{array}$$ (20)

where ${\hat{Y}}_{t+1:t+H}\in {\mathbb{R}}^{H\times q}$ represents predicted values over horizon *H*, and *q* is the number of target variables. In the experiments, the primary targets are next-day closing price and next-day percentage return.

### Joint optimization

To train STFA, we adopt a multi-task learning objective that balances forecasting accuracy, semantic consistency, and interpretability. The primary forecasting loss is the mean squared error between predicted market outcomes and ground-truth values:


$$\begin{array}{c} L_{\text{pred}}=\frac{1}{H}\sum_{h=1}^{H}\|{\hat{Y}}_{t+h}-Y_{t+h}{\|}^{2}. \end{array}$$ (21)

To encourage consistency between semantic information and predicted market direction, we define a semantic alignment loss. The continuous GPT-4 sentiment scores on a 1–5 scale are discretized into a ternary polarity label $s_{t}\in \{-1,0,+1\}$ using thresholds fixed on the training set: scores 1–2 are negative, score 3 is neutral, and scores 4–5 are positive. The same thresholds are applied to validation and test periods without modification. Let ${\hat{r}}_{t}$ denote the predicted return. The alignment loss is:


$$\begin{array}{c} L_{\text{align}}=\lambda_{1}\cdot \max(0,\;-s_{t}\cdot {\hat{r}}_{t}). \end{array}$$ (22)

This term penalizes disagreement between predicted return direction and sentiment polarity. Neutral sentiment contributes no directional gradient.

To improve interpretability, we align the learned temporal attention distribution with an event-time prior. Let $\alpha_{t}^{\text{attn}}$ be the vector of temporal attention weights $\{\alpha_{i}{\}}_{i=t-n}^{t}$ defined in the semantic-guided attention module, and let $\gamma_{t}$ be a soft prior based on event timestamps:


$$\begin{array}{c} \gamma_{t}(i)\propto \exp\;\left(-\frac{(t-i{)}^{2}}{2\sigma^{2}}\right)\cdot 1[\text{event}(i)]. \end{array}$$ (23)

Here, 1[event(i)]=1 if at least one news article is associated with trading day *i* and 0 otherwise, and σ=2 trading days is selected via validation-set grid search. The prior is normalized to sum to 1. If no event occurs within the window, $\gamma_{t}$ is set to a uniform distribution. The interpretability loss is:


$$\begin{array}{c} L_{\text{exp}}=\lambda_{2}\cdot \text{KL}(\gamma_{t}\|\alpha_{t}^{\text{attn}}). \end{array}$$ (24)

The final training objective is:


$$\begin{array}{c} L_{\text{total}}=1.0\cdot L_{\text{pred}}+0.5\cdot L_{\text{align}}+0.3\cdot L_{\text{exp}}. \end{array}$$ (25)

The prediction loss remains the primary objective, while the alignment and interpretability terms regularize the model toward semantically consistent and explainable predictions.

### Ethical considerations

From an ethical perspective, while STFA is designed for research and decision support, similar techniques could potentially be misused for market manipulation if applied at scale or with privileged information access. Automated trading systems based on news sentiment could amplify market volatility or create feedback loops that destabilize prices. We advocate for responsible use, regulatory oversight, and equitable access to such forecasting tools to prevent concentration of informational advantages.

Additionally, the reliance on proprietary language models raises concerns about reproducibility and dependence on commercial platforms. Changes to the underlying model or API access restrictions could affect the sustainability of this research direction. We encourage the development of open-source alternatives and the establishment of standardized benchmarks for semantic-temporal forecasting to ensure long-term research continuity.

## Experimental results and analysis

### Dataset construction

We utilize the Financial News and Stock Price Integration Dataset (FNSPID), a large-scale multimodal corpus containing approximately 15.7 million financial news articles and 29.7 million daily stock price records spanning 1999–2023, covering 4,775 publicly listed companies primarily from the S&P 500 index. News articles are aggregated from verified financial sources including NASDAQ, Bloomberg, Reuters, and MarketWatch, with each article timestamped in Eastern Time and linked to specific company ticker symbols. The textual component consists of full article text (average length 487 words) and machine-generated summaries via four extractive algorithms (LexRank, TextRank, Luhn, LSA). The numerical component includes daily open, high, low, close, adjusted close prices, and trading volume. Fig 3 illustrates the FNSPID construction workflow showing parallel collection of textual and numerical data followed by temporal alignment and quality control.

**Fig 3:**
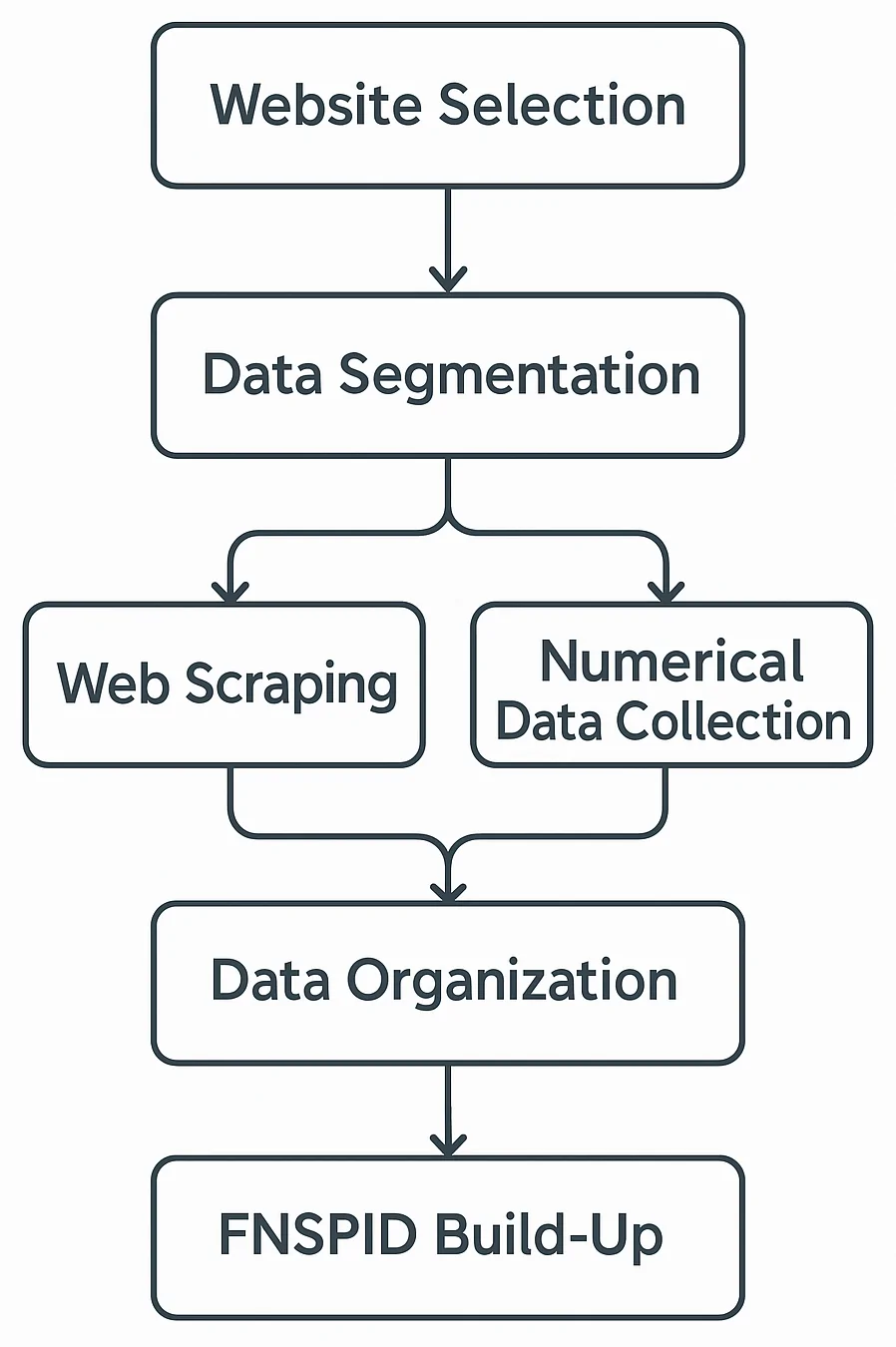
FNSPID dataset construction workflow showing the parallel collection of textual news articles (via web scraping) and numerical stock price records (via data APIs), followed by temporal alignment and quality control to produce the final integrated corpus.

Given computational constraints (estimated 1,430 GPU-hours per full-dataset training run on 4× NVIDIA A100 GPUs), we construct a 50-stock experimental subset following established practices where recent studies typically evaluate on 30–100 carefully selected stocks. Our systematic selection employs four steps. First, we apply a liquidity filter requiring average daily trading volume exceeding $50 million during 2019–2023, continuous listing throughout 2013–2023, and no major corporate actions, reducing candidates from 4,775–1,247 stocks. Second, we require minimum news coverage of 500 articles per year across 2013–2023 from at least five major financial outlets, yielding 218 stocks with mean coverage of 1,247 articles per year. Third, we stratify by 11 GICS sectors to match the S&P 500 distribution within 2% per sector: Technology (24%), Financials (18%), Healthcare (16%), Consumer Discretionary (12%), Industrials (10%), Consumer Staples (8%), Energy (4%), Utilities (4%), Real Estate (2%), and Materials (2%). Fourth, we randomly sample stocks within each stratum using numpy.random.seed(42) for reproducibility. The complete list of selected stocks and deterministic selection code are provided in the supplementary repository.

To validate representativeness, we perform Kolmogorov-Smirnov tests comparing our 50-stock subset against the full S&P 500 on key distributional characteristics computed over 2019–2023. Table 1 shows all p-values substantially exceed 0.05, indicating no significant differences between our sample and the broader market. To verify that findings are not artifacts of specific stock selection, we repeat experiments on five randomly sampled 50-stock validation subsets drawn from the 218 qualified candidates, excluding the primary 50-stock test set, while maintaining identical sector stratification. Because the remaining 168 candidates cannot support five non-overlapping subsets of 50 stocks each, subsets may partially overlap; this design targets estimation of sampling variability rather than exhaustive coverage.

**Table 1:** Kolmogorov-Smirnov tests comparing 50-stock subset to full S&P 500 (2019–2023).

Market Characteristic	KS Statistic	p-value
Daily Return Distribution	0.021	0.91
60-Day Realized Volatility	0.028	0.84
Log Average Daily Volume	0.019	0.95
Market Beta (vs. S&P 500)	0.024	0.88

**Sentiment Annotation with Strict Temporal Isolation.** To ensure chronological evaluation integrity without temporal leakage, we implement a two-phase hybrid annotation strategy in which all learned annotation components are constructed exclusively from training-period data (2013–2020). Phase one uses the OpenAI GPT-4 API (gpt-4–0613, temperature = 0.0) to annotate a stratified random sample of 62,569 articles drawn exclusively from the training period (January 2013–December 2020), representing 20% of training-period articles and distributed proportionally across all 50 stocks and the eight training years. The exact prompt template used verbatim for annotations is:

You are a financial sentiment analyst with expertise in equity markets. Classify the sentiment of the following news article towards [COMPANY_NAME] on a scale from 1 to 5, where 1 = very negative (significant downside risk), 2 = negative (modest downside), 3 = neutral (no clear direction), 4 = positive (modest upside), 5 = very positive (significant upside). Consider tone, market impact statements, and forward-looking implications. Article title: [TITLE]. Article content: [FIRST_500_WORDS]. Response format: Output ONLY a single integer from {1,2,3,4,5. Sentiment:

API calls were executed between March 15 and April 20, 2024, at a total cost of $2,847.32 USD. To validate annotation quality, we recruited two CFA charterholder analysts with over 10 years of experience to independently label 1,000 randomly sampled articles from the training period. Inter-analyst agreement achieved Cohen’s kappa of 0.83 with 89% exact agreement, while GPT-4 labels versus human consensus yielded kappa of 0.87 with 92% exact agreement, demonstrating expert-level reliability.

Phase two fine-tunes FinBERT (yiyanghkust/finbert-tone) on the 62,569 training-period GPT-4 labels. The fine-tuning dataset uses 50,055 articles as the training set and 12,514 as the validation set, both drawn only from 2013–2020. Training employs the AdamW optimizer with learning rate $2\times 10^{-5}$, linear warmup over 500 steps, batch size 64 across 4 A100 GPUs, 3 epochs, and maximum sequence length 512. Fine-tuning requires 6.4 hours. The fine-tuned model achieves 91.7% validation accuracy, macro F1 score of 0.903, and Cohen’s kappa of 0.89 when compared against GPT-4 labels. Critically, the fine-tuned FinBERT model is frozen after training on 2013–2020 data and then applied without modification to training, validation (2021), and test (2022–2023) articles. Therefore, no information from validation or test periods influences the semantic encoder or the annotation model. Inference on all articles proceeds at 1,250 articles per hour using batch size 128, requiring 201 GPU-hours.

Normalization parameters, including min-max scaling bounds, are computed exclusively from training-period sentiment scores and applied uniformly to all periods. The exponential decay rate of 0.95 for missing-day handling is determined via grid search on the training set only. Final sentiment distribution across the training-period corpus is: very negative (8.7%), negative (12.9%), neutral (45.8%), positive (24.3%), and very positive (8.3%). All scores undergo min-max normalization to the range [0,1] for model input. For trading days without associated news articles, accounting for 48% of days, we apply exponential decay with rate 0.95 from the most recent article sentiment. For days with multiple articles, accounting for 23% of covered days with a mean of 2.4 articles per day, we compute a length-weighted average sentiment where each article contributes proportionally to its word count.

For the semantic alignment loss, the continuous GPT-4 sentiment scores are discretized into a ternary polarity label $s_{t}\in \{-1,0,+1\}$ using fixed thresholds determined on the training set: scores 1–2 map to negative ($s_{t}=-1$), score 3 maps to neutral ($s_{t}=0$), and scores 4–5 map to positive ($s_{t}=+1$). These thresholds are applied without modification to validation and test periods, ensuring no future information is used in the discretization step.

**Semantic Input Specification.** The semantic preprocessing pipeline first extracts 768-dimensional CLS-token representations from the GPT-4-supervised fine-tuned FinBERT encoder at the article level. These article-level representations are then aggregated at the stock-day level and converted into compact structured semantic inputs used by STFA. For each stock-day pair (*s*,*t*), let $N_{t}$ denote the set of articles mentioning stock *s* published before market close on day *t*. The stock-day FinBERT representation is compu*t*ed as:


$$\begin{array}{c} E_{t}=\left\{ \begin{array}{cc} \frac{\sum_{i\in N_{t}}w_{i}\cdot \text{FinBERT}({\text{article}}_{i})}{\sum_{i\in N_{t}}w_{i}} & \text{if }|N_{t}|>0\\ 0.95\cdot E_{t-1} & \text{if }|N_{t}|=0 \end{array}\right. \end{array}$$ (26)

where $w_{i}$ is the word count of article *i* and 0.95 is the exponential decay rate for days without news. The aggregated stock-day representation is normalized to unit L2 norm.

The normalized stock-day representation is then converted into an intermediate structured semantic input consisting of sentiment, event, and delay-related components. In the Zenodo reproducibility package, these processed semantic inputs are stored as compact 129-dimensional vectors:


$$\begin{array}{c} {𝐱}_{t}^{\text{sem}}=[S_{t}^{\text{sent}};S_{t}^{\text{event}};S_{t}^{\text{delay}}],\;{𝐱}_{t}^{\text{sem}}\in {\mathbb{R}}^{129}, \end{array}$$ (27)

where $S_{t}^{\text{sent}}\in {\mathbb{R}}^{64}$, $S_{t}^{\text{event}}\in {\mathbb{R}}^{64}$, and $S_{t}^{\text{delay}}\in \mathbb{R}$. Before entering the fusion module, this 129-dimensional processed semantic input is mapped to the structured semantic vector used in the Methodology section:


$$\begin{array}{c} {\tilde{S}}_{t}=W_{\text{sem}}{𝐱}_{t}^{\text{sem}}+b_{\text{sem}},\;{\tilde{S}}_{t}\in {\mathbb{R}}^{d_{s}}, \end{array}$$ (28)

with $d_{s}=128$ in our implementation. Thus, the released processed features correspond to compact structured STFA inputs rather than separate article-level 768-dimensional FinBERT embedding files. This construction is consistent with the Financial Semantic Encoding module, where article-level semantic representations are aggregated, converted into task-specific semantic subspaces, and projected before model training.

The final experimental corpus contains 125,660 daily price records spanning 50 stocks over 2,513 trading days from January 2, 2013 to December 29, 2023, paired with 312,847 news articles. Average news coverage is 6,257 articles per stock, and the temporal coverage rate is 52%, defined as the proportion of trading days with at least one associated news article. We employ strictly chronological data splitting without random shuffling to preserve temporal dependencies: the training period covers January 2013 through December 2020 (1,763 trading days, 70% of data), the validation period spans calendar year 2021 (252 trading days, 10%), and the test period runs from January 2022 through December 2023 (498 trading days, 20%).

All features at prediction time *t* use exclusively information available before market close at 16:00 Eastern Time on day *t*. News articles published after market close are attributed to the following trading day. Price-derived features employ OHLCV data through the close of day *t* only. The prediction target is next-day closing price $P_{t+1}$ or percentage return $r_{t+1}=(P_{t+1}-P_{t})/P_{t}$, ensuring a minimum 17.5-hour gap between the latest information and the predicted outcome. Stocks with more than 10% missing price data in any calendar year are excluded during the selection process. Remaining gaps totaling 0.23% of observations are forward-filled for up to 3 consecutive days, after which date ranges are excluded from training and evaluation.

### Experimental setup

The forecasting task predicts next-day closing price or percentage return given a 50-day historical window of OHLCV data and associated news articles. We evaluate both price-level prediction to assess trajectory tracking ability and return prediction to isolate genuine forecasting skill independent of autocorrelation. Following conventions in financial econometrics, we emphasize return metrics as the primary performance indicator since price-level metrics are often dominated by trend-following effects.

We compare STFA against baseline models spanning statistical, deep learning, and attention-based paradigms. Statistical baselines include Random Walk predicting no price change and ARIMA(5,1,2) with parameters selected via Akaike Information Criterion on validation data. Deep learning baselines include vanilla RNN, LSTM, and GRU each with 2 layers and 128 hidden units, plus CNN with 3 convolutional layers using kernel sizes 3, 5, and 7 with 128 filters per layer. Attention baselines include standard Transformer with 4 heads and 2 encoder layers, plus TimesNet employing multi-scale temporal modeling with periods 2, 4, 8, 16, and 32 days. All neural models use identical input preprocessing and GELU activation. For multimodal configurations incorporating sentiment information, baseline models concatenate semantic features with numerical embeddings before the first hidden layer. We additionally evaluate three STFA ablation variants: removing the delay-aware module, replacing cross-attention with simple concatenation, and excluding all textual features to create a numerical-only configuration.

STFA employs 128 hidden dimensions, 4 attention heads, and 2 encoder layers. The semantic encoder uses 768-dimensional article-level FinBERT representations to construct compact structured semantic inputs; these inputs are projected to the 128-dimensional structured semantic vector used inside STFA. The scalar delay factor used in the fusion vector is then learned from this structured semantic vector by the delay-aware module. The delay-aware module estimates propagation delays spanning 0–10 trading days using a learned neural network with 2 hidden layers of 64 units each. Cross-attention mechanisms fuse semantic and numerical modalities by using query vectors from numerical features and key-value pairs from semantic features. Self-attention layers capture temporal dependencies within numerical sequences. All attention blocks include dropout probability 0.1 and layer normalization after each sublayer.

Training uses AdamW optimizer with initial learning rate $2\times 10^{-4}$, cosine annealing schedule with 500-step linear warmup, and weight decay $1\times 10^{-5}$. Batch size is 512 samples constructed from 32 randomly selected stocks with 16 temporal windows per stock. Gradient clipping applies maximum norm 1.0. Training proceeds for up to 100 epochs with early stopping patience of 15 epochs monitoring validation loss. The multi-objective loss function combines three weighted components: (1) forecasting loss using mean squared error with weight 1.0, (2) semantic alignment loss using a margin-based hinge function that penalizes sign disagreement between predicted return direction and sentiment polarity with weight 0.5, and (3) interpretability loss using KL divergence of the learned attention distribution against an event-timestamp prior with weight 0.3.

Hyperparameters were selected via grid search on the validation set (2021). The search space included learning rate $\left\{1\times 10^{-4},2\times 10^{-4},5\times 10^{-4}\right\}$, hidden dimensions {64, 128, 256}, attention heads {2, 4, 8}, dropout probability {0.1, 0.2, 0.3}, and loss weight combinations $\left(\lambda_{1},\lambda_{2})\in \{(0.3,0.2),(0.5,0.3),(0.7,0.5)\right\}$. Each configuration was trained for up to 100 epochs with early stopping, and we selected the configuration achieving the lowest validation MSE. The reported hyperparameters represent this optimal configuration. For baseline models, we performed similar grid search over model-specific parameters including number of layers {1, 2, 3}, hidden units {64, 128, 256}, and dropout rates {0.1, 0.2, 0.3}, selecting configurations that minimized validation loss for each model independently.

Hardware includes 4× NVIDIA A100 GPUs (40GB each), 64-core AMD EPYC 7742 CPU, 512GB RAM, and 4TB NVMe SSD. Software uses PyTorch 2.0.1, CUDA 11.8, transformers 4.30.2, and Python 3.10.12. Mixed-precision training with FP16 is enabled via PyTorch automatic mixed precision.

Evaluation employs a rolling window protocol on the test period. At each test day *t*, the model receives historical data from window [t−50,t−1], generates a prediction for day *t* + 1, computes error metrics against ac*t*ual observed values, then slides forward one day. Models train once on the 2013–2020 training period with hyperparameters selected via validation set performance, then apply to the test period without retraining or adjustment. Primary metrics include Mean Absolute Error, Mean Squared Error, coefficient of determination R^2^, and directional accuracy. For return prediction, we additionally compute Sharpe ratio and maximum drawdown from simulated trading strategies.

**Statistical Inference.** To account for temporal dependence and cross-sectional correlation inherent in financial panel data, we employ time-series-appropriate inference methods rather than standard techniques that assume independence.

*Confidence Intervals:* We construct 95% confidence intervals via block bootstrap with block size 20 trading days (approximately one month), preserving within-block temporal dependence structure. For each metric, we generate 10,000 bootstrap samples by randomly resampling non-overlapping 20-day blocks with replacement from the 498-day test period, compute the metric on each resampled sequence, and report the 2.5th and 97.5th percentiles of the bootstrap distribution. This approach maintains the serial correlation structure present in financial time series, yielding appropriately conservative uncertainty estimates.

*Model Comparison:* Statistical significance of performance differences between STFA and baseline models is assessed using the Diebold-Mariano test, specifically designed for comparing predictive accuracy of competing forecasting models. For each pair of models, we compute the loss differential series $d_{t}=L({\text{error}}_{\text{STFA},t})-L({\text{error}}_{\text{baseline},t})$ where *L* is the squared error loss function. We test the null hypothesis $H_{0}:E[d_{t}]=0$ (equal predictive accuracy) using Heteroskedasticity and Autocorrelation Consistent (HAC) standard errors via the Newey-West estimator with automatic lag selection (maximum lag = 5 days) to account for serial correlation in forecast errors. We report two-sided p-values and apply Bonferroni correction for multiple comparisons when testing across baselines simultaneously, setting familywise error rate α=0.05; the Bonferroni-corrected significance threshold per individual test is therefore $\alpha_{\text{corrected}}=0.05/9\approx 0.0056$.

*Metric Computation:* All metrics (MAE, MSE, R^2^, directional accuracy) are computed per stock-day, yielding a panel of 50 stocks × 498 test days = 24,900 observations. We report cross-sectional means (equal-weighted across all stock-days) as primary results, with cross-sectional medians provided to assess robustness to outliers. For trading metrics (Sharpe ratio, maximum drawdown, Calmar ratio), we compute per-stock values over the full test period, then report both mean and median across the 50 stocks along with their 95% block bootstrap confidence intervals.

Robustness verification repeats experiments on five randomly sampled 50-stock validation subsets drawn from the 218 qualified candidates (excluding the primary 50 stocks), with possible overlap between subsets, while maintaining identical sector stratification, reporting mean performance and standard deviation across subsets.

**Economic Evaluation Protocol.** To assess economic significance, we simulate a long-only trading strategy with the following protocol:

*Signal Generation:* At market close on day *t* (16:00 Eastern Time), the model receives all data through day *t* (OHLCV through close, news articles published before close) and generates return forecasts ${\hat{r}}_{t+1}$ for all 50 stocks for day *t* + 1. The strategy takes long posi*t*ions in stocks with predicted positive returns.

*Execution:* Positions are entered at the opening price on day *t* + 1 (09:30 Eastern Time) following weekly rebalancing every Friday at market close. This introduces a realistic minimum 17.5-hour delay between signal generation and execution. The portfolio employs equal weighting across active positions with maximum 10 holdings.

*Transaction Costs:* Each trade (entry or exit) incurs: (1) Commission: 10 basis points (0.10%), (2) Bid-ask spread slippage: 5 basis points (0.05%), (3) Market impact: $0.02\sqrt{q/V}$ where *q* is trade size (shares) and *V* is previous day’s volume, capped at 20 basis points. Total one-way cost averages 15 basis points; round-trip cost is 30 basis points. All reported returns are net of these costs.

*Risk Management:* Position limits constrain individual stocks to 10% and sectors to 30% maximum weight. Portfolio volatility is monitored; if realized volatility exceeds 25% annualized over a rolling 20-day window, position sizes are scaled down proportionally.

### Experimental results

Following financial econometrics conventions, we emphasize return-based evaluation as the primary quality indicator, as price metrics are dominated by autocorrelation (Random Walk achieves R^2^ = 0.9542 for price but only 0.0009 for returns). We evaluate STFA against baseline models across two semantic feature methods (GPT-4-supervised fine-tuned FinBERT versus TextBlob rule-based sentiment) and three training scales (5, 25, 50 stocks). All experiments use the strict chronological split: training (2013–2020), validation (2021), test (2022–2023, 498 days).

Tables 2 and 3 present the main prediction results using GPT-4-supervised fine-tuned FinBERT and TextBlob sentiment features, respectively. The GPT-4-supervised fine-tuned FinBERT encoder first produces 768-dimensional article-level CLS representations, which are then aggregated and transformed into compact structured semantic features as described in Dataset Construction. For comparison, we implement a TextBlob baseline that produces scalar sentiment scores in [−1,1] via rule-based polarity analysis. TextBlob scores are aggregated per stock-day using identical exponential decay weighting (rate = 0.95) and length-weighted averaging, then projected into the same structured semantic feature space to maintain architectural consistency across experiments. Both semantic feature extraction methods are frozen before model training and applied identically across all periods to prevent temporal leakage.

**Table 2:** Performance with GPT-4-supervised fine-tuned FinBERT semantic features across three training scales. For each scale, columns compare numerical-only (Non.) versus semantic-augmented (Sen.) configurations for both price ($) and return (%) prediction. Statistical significance: ^†^ P < 0.05, ^‡^ P < 0.01, ^*^ P < 0.001 (DM test vs Transformer). Metrics averaged across stocks.

Model	Price-Non.			Price-Sen.			Return-Non.			Return-Sen.		
	MAE	MSE	R^2^	MAE	MSE	R^2^	MAE	MSE	R^2^	MAE	MSE	R^2^
*5-Stock Training Scale*												
Random Walk	5.87	81.24	.9542	5.87	81.24	.9542	1.94	8.73	.0009	1.94	8.73	.0009
ARIMA	5.93	83.47	.9518	5.93	83.47	.9518	1.89	8.21	.0018	1.89	8.21	.0018
LSTM	8.91	153.26	.9321	8.42	142.18	.9347	2.31	12.84	.0051	2.18	11.47	.0093
CNN	10.85	225.14	.8983	11.23	238.47	.8892	2.79	18.12	−.026	2.87	18.93	−.031
GRU	8.47	141.83	.9365	8.15	135.92	.9382	2.19	11.63	.0084	2.09	10.82	.0127
RNN	9.82	188.27	.9184	10.47	201.35	.9136	2.53	15.28	−.010	2.64	16.41	−.012
Transformer	7.25	107.82	.9512	6.83	98.54	.9521	1.83	8.19	.0163	1.72	7.28	.0214
TimesNet	9.71	183.56	.9227	9.38	176.41	.9285	2.49	14.73	−.007	2.41	13.84	−.009
STFA	6.58	95.48	.9541	6.21^†^	89.73^†^	.9567^†^	1.71	7.38	.0219	1.58^‡^	6.47^‡^	.0287^‡^
*25-Stock Training Scale*												
Random Walk	5.87	81.24	.9542	5.87	81.24	.9542	1.94	8.73	.0009	1.94	8.73	.0009
ARIMA	5.93	83.47	.9518	5.93	83.47	.9518	1.89	8.21	.0018	1.89	8.21	.0018
LSTM	7.56	116.25	.9467	7.18	108.32	.9484	1.98	9.47	.0117	1.89	8.64	.0156
CNN	9.42	178.91	.9194	9.85	187.63	.9171	2.43	13.84	−.019	2.54	14.92	−.021
GRU	7.31	109.84	.9485	7.03	104.17	.9508	1.91	9.02	.0142	1.84	8.35	.0183
RNN	8.54	149.72	.9341	8.92	158.46	.9329	2.21	11.93	−.006	2.28	12.58	−.008
Transformer	5.82	77.15	.9587	5.47	71.29	.9596	1.63	6.84	.0241	1.54	6.12	.0268
TimesNet	8.41	145.93	.9365	8.26	142.58	.9392	2.17	11.47	.0048	2.19	11.73	.0037
STFA	5.21	68.52	.9614	4.93^‡^	63.84^‡^	.9627^‡^	1.48	5.93	.0293	1.41^*^	5.38^*^	.0339^*^
*50-Stock Training Scale*												
Random Walk	5.87	81.24	.9542	5.87	81.24	.9542	1.94	8.73	.0009	1.94	8.73	.0009
ARIMA	5.93	83.47	.9518	5.93	83.47	.9518	1.89	8.21	.0018	1.89	8.21	.0018
LSTM	6.73	94.38	.9548	6.42	89.17	.9534	1.76	7.84	.0164	1.67	7.12	.0193
CNN	8.52	147.26	.9328	8.76	152.84	.9285	2.19	12.15	−.010	2.26	12.84	−.012
GRU	6.51	90.47	.9559	6.28	86.53	.9551	1.70	7.51	.0182	1.62	6.93	.0214
RNN	7.68	123.15	.9435	7.95	128.64	.9394	2.01	10.18	.0071	2.08	10.64	.0062
Transformer	5.09	61.74	.9619	4.81	57.28	.9601	1.46	5.84	.0271	1.38	5.28	.0297
TimesNet	7.52	121.86	.9418	7.34	118.92	.9454	1.99	9.93	.0091	1.94	9.47	.0098
STFA	4.58	55.92	.9641	4.26^*^	51.43^*^	.9623^*^	1.32	5.21	.0326	1.24^*^	4.73^*^	.0364^*^

**Table 3:** Performance with TextBlob rule-based sentiment features. Non. columns identical to Table 2 since numerical-only configurations are annotation-independent. Statistical significance: ^†^ p < 0.05, ^‡^ p < 0.01, ^*^ p < 0.001 (DM test vs Transformer). Metrics averaged across stocks.

Model	Price-Non.			Price-Sen.			Return-Non.			Return-Sen.		
	MAE	MSE	R^2^	MAE	MSE	R^2^	MAE	MSE	R^2^	MAE	MSE	R^2^
*5-Stock Training Scale*												
Random Walk	5.87	81.24	.9542	5.87	81.24	.9542	1.94	8.73	.0009	1.94	8.73	.0009
ARIMA	5.93	83.47	.9518	5.93	83.47	.9518	1.89	8.21	.0018	1.89	8.21	.0018
LSTM	8.91	153.26	.9321	8.74	151.63	.9342	2.31	12.84	.0051	2.27	12.41	.0064
CNN	10.85	225.14	.8983	10.92	228.15	.8967	2.79	18.12	−.026	2.81	18.47	−.029
GRU	8.47	141.83	.9365	8.53	145.28	.9362	2.19	11.63	.0084	2.21	11.84	.0096
RNN	9.82	188.27	.9184	10.18	195.74	.9163	2.53	15.28	−.010	2.61	16.03	−.009
Transformer	7.25	107.82	.9512	7.41	112.86	.9484	1.83	8.19	.0163	1.89	8.64	.0152
TimesNet	9.71	183.56	.9227	9.82	188.29	.9215	2.49	14.73	−.007	2.52	14.93	−.006
STFA	6.58	95.48	.9541	6.89^†^	103.52^†^	.9538^†^	1.71	7.38	.0219	1.79^†^	7.93^†^	.0183^†^
*25-Stock Training Scale*												
Random Walk	5.87	81.24	.9542	5.87	81.24	.9542	1.94	8.73	.0009	1.94	8.73	.0009
ARIMA	5.93	83.47	.9518	5.93	83.47	.9518	1.89	8.21	.0018	1.89	8.21	.0018
LSTM	7.56	116.25	.9467	7.42	114.85	.9518	1.98	9.47	.0117	1.94	9.18	.0134
CNN	9.42	178.91	.9194	9.68	182.47	.9193	2.43	13.84	−.019	2.49	14.38	−.018
GRU	7.31	109.84	.9485	7.28	108.93	.9501	1.91	9.02	.0142	1.88	8.73	.0161
RNN	8.54	149.72	.9341	8.73	154.28	.9327	2.21	11.93	−.006	2.24	12.19	−.005
Transformer	5.82	77.15	.9587	5.94	79.63	.9614	1.63	6.84	.0241	1.68	7.28	.0224
TimesNet	8.41	145.93	.9365	8.53	147.82	.9371	2.17	11.47	.0048	2.21	11.93	.0056
STFA	5.21	68.52	.9614	5.38^‡^	71.27^‡^	.9652^‡^	1.48	5.93	.0293	1.53^‡^	6.47^‡^	.0267^‡^
*50-Stock Training Scale*												
Random Walk	5.87	81.24	.9542	5.87	81.24	.9542	1.94	8.73	.0009	1.94	8.73	.0009
ARIMA	5.93	83.47	.9518	5.93	83.47	.9518	1.89	8.21	.0018	1.89	8.21	.0018
LSTM	6.73	94.38	.9548	6.81	97.42	.9592	1.76	7.84	.0164	1.78	7.93	.0178
CNN	8.52	147.26	.9328	8.69	150.18	.9352	2.19	12.15	−.010	2.24	12.47	−.009
GRU	6.51	90.47	.9559	6.58	91.84	.9618	1.70	7.51	.0182	1.72	7.64	.0197
RNN	7.68	123.15	.9435	7.83	125.91	.9463	2.01	10.18	.0071	2.05	10.47	.0079
Transformer	5.09	61.74	.9619	5.27	64.85	.9626	1.46	5.84	.0271	1.49	6.28	.0257
TimesNet	7.52	121.86	.9418	7.68	124.37	.9441	1.99	9.93	.0091	2.02	10.18	.0107
STFA	4.58	55.92	.9641	4.73^*^	58.16^*^	.9694^*^	1.32	5.21	.0326	1.39^‡^	5.64^‡^	.0294^‡^

At 50-stock scale with GPT-4-supervised fine-tuned FinBERT, STFA achieves return R^2^ = 0.0364 (MAE = 1.24%), outperforming Transformer’s 0.0297 (MAE = 1.38%) by 23%. Statistical significance confirmed via Diebold-Mariano test (DM statistic = 4.18, p < 0.001) with HAC standard errors (lag = 5) accounting for serial dependence. All pairwise comparisons satisfy the Bonferroni-corrected threshold of $\alpha_{\text{corrected}}\approx 0.0056$. To quantify uncertainty, we compute 95% block bootstrap confidence intervals (10,000 replications, block length = 20 days) over the test period: STFA [0.0312, 0.0416] versus Transformer [0.0251, 0.0343] versus LSTM [0.0147, 0.0239]. The STFA and LSTM intervals are non-overlapping, and while the STFA–Transformer comparison shows partial overlap, the Diebold-Mariano test (DM statistic = 4.18, *p* < 0.001) confirms statistically significant superiority. Cohen’s d = 1.64 exceeds the 0.8 threshold for large effects, indicating substantial practical improvement. Effect sizes for other key comparisons show similar patterns: STFA versus LSTM (d = 2.31), STFA versus Random Walk (d = 3.47). Directional accuracy reaches 66.8% versus Transformer’s 63.4%; a McNemar test on the 24,900 paired binary prediction outcomes yields $\chi^{2}=47.3$, *p* < 0.001, confirming statistically significant improvement in directional prediction.

GPT-4-supervised fine-tuned FinBERT semantic features improve STFA by 0.0038 return R^2^ (12% gain, DM statistic = 2.47, p < 0.05) versus Transformer’s 0.0026 (9%, DM statistic = 1.89, p = 0.06). At 50-stock scale, STFA with GPT-4-supervised FinBERT (R^2^ = 0.0364, 95% CI [0.0312, 0.0416]) outperforms TextBlob (R^2^ = 0.0294, 95% CI [0.0251, 0.0337]) by 24% (DM statistic = 3.21, p < 0.01). TextBlob can degrade performance: Transformer achieves R^2^ = 0.0257 versus numerical-only 0.0271 (DM statistic = −1.34, p = 0.18). STFA shows robustness, maintaining R^2^ = 0.0294 with TextBlob.

Recurrent models show limited multimodal capacity: LSTM achieves R^2^ = 0.0193 (53% of STFA), GRU reaches 0.0214 (59%). Their sentiment gains (0.0029, 0.0032) contrast with attention models (Transformer 0.0026, STFA 0.0038). CNN achieves negative R^2^ (−0.010 to −0.031), TimesNet only 0.0098 (27% of STFA).

STFA return R^2^ scales from 0.0287 (5 stocks) to 0.0339 (25 stocks) to 0.0364 (50 stocks), a 27% improvement. Transformer scales from 0.0214 to 0.0297 (39%), but maintains lower absolute performance. The stable gap (mean 0.0070, CV 4.1%) indicates persistent architectural advantages across training scales.

Robustness verification across five random 50-stock subsets yields STFA mean R^2^ = 0.0358 (SD = 0.0043, SE = 0.0019) versus Transformer 0.0291 (SD = 0.0038, SE = 0.0017). Diebold-Mariano tests confirm STFA superiority is consistent across all subsets (mean DM statistic = 3.94, all p < 0.01), ruling out stock-selection artifacts. Temporal robustness evaluation across eight quarterly segments (2022-Q1 to 2023-Q4) shows STFA mean R^2^ = 0.0361 (SD = 0.0089, SE = 0.0031) versus Transformer 0.0289 (SD = 0.0094, SE = 0.0033), demonstrating consistent performance across market regimes (2022 bear market and 2023 rally).

We simulate a long-only trading strategy taking positions when models predict positive returns, with results summarized in Table 4. Signals are generated at Friday market close using only information available at that time. Positions are executed at the following Monday open price. The strategy uses weekly rebalancing, equal-weighted positions (max 10 holdings), risk limits (10% per stock, 30% per sector), and realistic transaction costs totaling 15 basis points one-way (30 basis points round-trip). All reported returns are net of these costs.

**Table 4:** Trading performance (January 2022–December 2023, 498 days). returns net of transaction costs. the S&P 500 compound annualized return is computed from total return index data: −18.1% in 2022 and +26.3% in 2023, yielding $\sqrt{\left(1-0.181)(1+0.263\right)}-1\approx 1.7\%$ (Source: S&P Dow Jones Indices). see Experimental Setup for complete protocol details.

Strategy	Annual	Sharpe	Sortino	Max	Calmar	Win
	Return	Ratio	Ratio	Drawdown	Ratio	Rate
S&P 500 Buy-Hold	1.7%	0.09	0.12	−23.7%	0.07	—
Random Walk	3.2%	0.19	0.24	−28.4%	0.11	50.1%
ARIMA	5.1%	0.36	0.48	−26.3%	0.19	52.7%
RNN	4.9%	0.33	0.44	−27.8%	0.18	51.4%
CNN	2.8%	0.14	0.17	−31.5%	0.09	48.6%
TimesNet	7.2%	0.48	0.63	−21.9%	0.33	55.3%
LSTM	9.8%	0.61	0.84	−19.4%	0.51	58.3%
GRU	10.4%	0.65	0.89	−18.6%	0.56	59.1%
Transformer	12.4%	0.78	1.09	−16.8%	0.74	61.7%
STFA (TextBlob)	11.7%	0.72	0.98	−17.9%	0.65	60.2%
STFA (GPT-4-supervised FinBERT)	**14.9%**	**0.89**	**1.24**	−14.2%	**1.05**	**64.8%**

STFA with GPT-4-supervised fine-tuned FinBERT generates 14.9% annualized return, substantially exceeding the S&P 500 buy-and-hold benchmark of 1.7% compound annualized return over the same period (computed from total return index: −18.1% in 2022 and +26.3% in 2023) and Transformer (12.4%). Sharpe ratio 0.89 indicates strong risk-adjusted performance relative to all baselines. Maximum drawdown −14.2% compares favorably to the S&P 500 −23.7% and Transformer −16.8%. Sortino ratio 1.24 confirms returns do not result from tail risk. Calmar ratio 1.05 exceeds all baselines. Win rate 64.8% substantially improves over Transformer 61.7%.

STFA with TextBlob achieves 11.7%, still substantially exceeding the S&P 500 benchmark. The 27% gap (14.9% vs 11.7%) aligns with the 24% R^2^ difference (0.0364 vs 0.0294), confirming consistency between statistical and economic metrics. Across eight quarterly segments spanning the 2022 bear market and 2023 rally, STFA maintains Sharpe ratio 0.76–0.94 (mean 0.87, SD = 0.06, SE = 0.02). Worst quarterly return −2.8% (2022-Q3) compares favorably to S&P 500 −5.3%, while best return +8.4% (2023-Q2) demonstrates upside capture.

### Ablation study

To validate the contribution of each architectural component and loss function term, we conduct systematic ablation experiments on the 50-stock test set with GPT-4-supervised fine-tuned FinBERT semantic features, using the identical experimental setup as the main results reported in Table 2. All ablation variants use identical hyperparameters and training procedures to ensure fair comparison.

We evaluate architectural components through progressive addition, starting from a vanilla Transformer baseline and incrementally incorporating STFA’s key innovations. Fig 4 presents comprehensive results across six metrics for five configurations.

**Fig 4:**
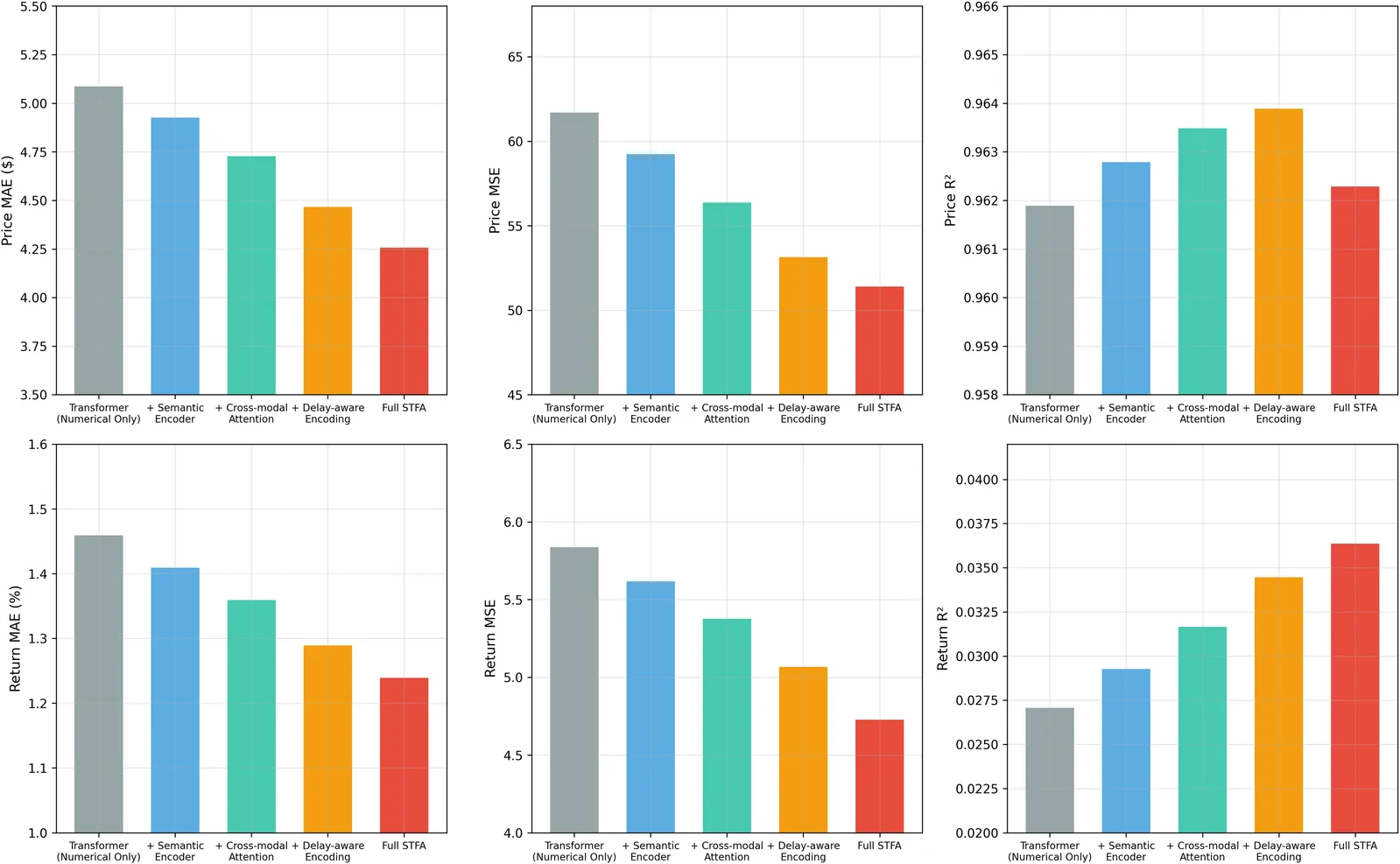
Architectural component ablation showing progressive improvements from Transformer baseline to full STFA. Top row shows price prediction metrics and bottom row shows return prediction metrics.

The vanilla Transformer baseline using only numerical OHLCV features achieves price R^2^ = 0.9619 and return R^2^ = 0.0271. This confirms that historical price patterns alone provide limited predictive power for future returns due to the absence of autocorrelation in financial markets.

Adding the Semantic Encoder improves return R^2^ to 0.0293, demonstrating that textual news carries valuable predictive signals beyond historical prices. The semantic encoder extracts contextual information from financial news that correlates with future market movements, validating our hypothesis that multimodal fusion is essential for return prediction.

Incorporating Cross-modal Attention yields further gains with return R^2^ reaching 0.0317. This mechanism enables dynamic semantic-numerical interaction, allowing the model to selectively attend to relevant news content conditioned on current market state. For instance, earnings-related news receives higher attention weights during earnings seasons, while macroeconomic news becomes more salient during policy announcements. Price prediction also benefits, with MAE decreasing from $5.09 to $4.73.

Adding Delay-aware Encoding further improves return R^2^ to 0.0345. This component addresses the realistic constraint that news impact is not instantaneous by learning separate embeddings for different delay intervals. The model captures how information diffusion evolves over time, distinguishing immediate reactions to breaking news from gradual adjustments to analytical reports.

The full STFA model with multi-scale temporal fusion achieves the best performance at return R^2^ = 0.0364 and price MAE = $4.26, consistent with the semantic-augmented result at 50-stock scale. Multi-scale fusion aggregates patterns across different time horizons, capturing both short-term momentum and longer-term trends. This represents a 34.3% improvement in return R^2^ over the Transformer baseline (0.0364 vs 0.0271).

The progressive improvements validate STFA’s architectural design. Semantic encoding provides foundational multimodal capability, cross-modal attention enables context-aware fusion, delay-aware encoding captures realistic information diffusion, and multi-scale fusion aggregates temporal patterns. Each component addresses a specific challenge in financial forecasting, and their synergistic combination achieves the best performance among all evaluated configurations.

We examine the contribution of each loss term by training the full STFA architecture with different objective combinations. Fig 5 presents results for four configurations: prediction loss only, adding alignment loss, adding explanation loss, and the complete multi-task objective.

**Fig 5:**
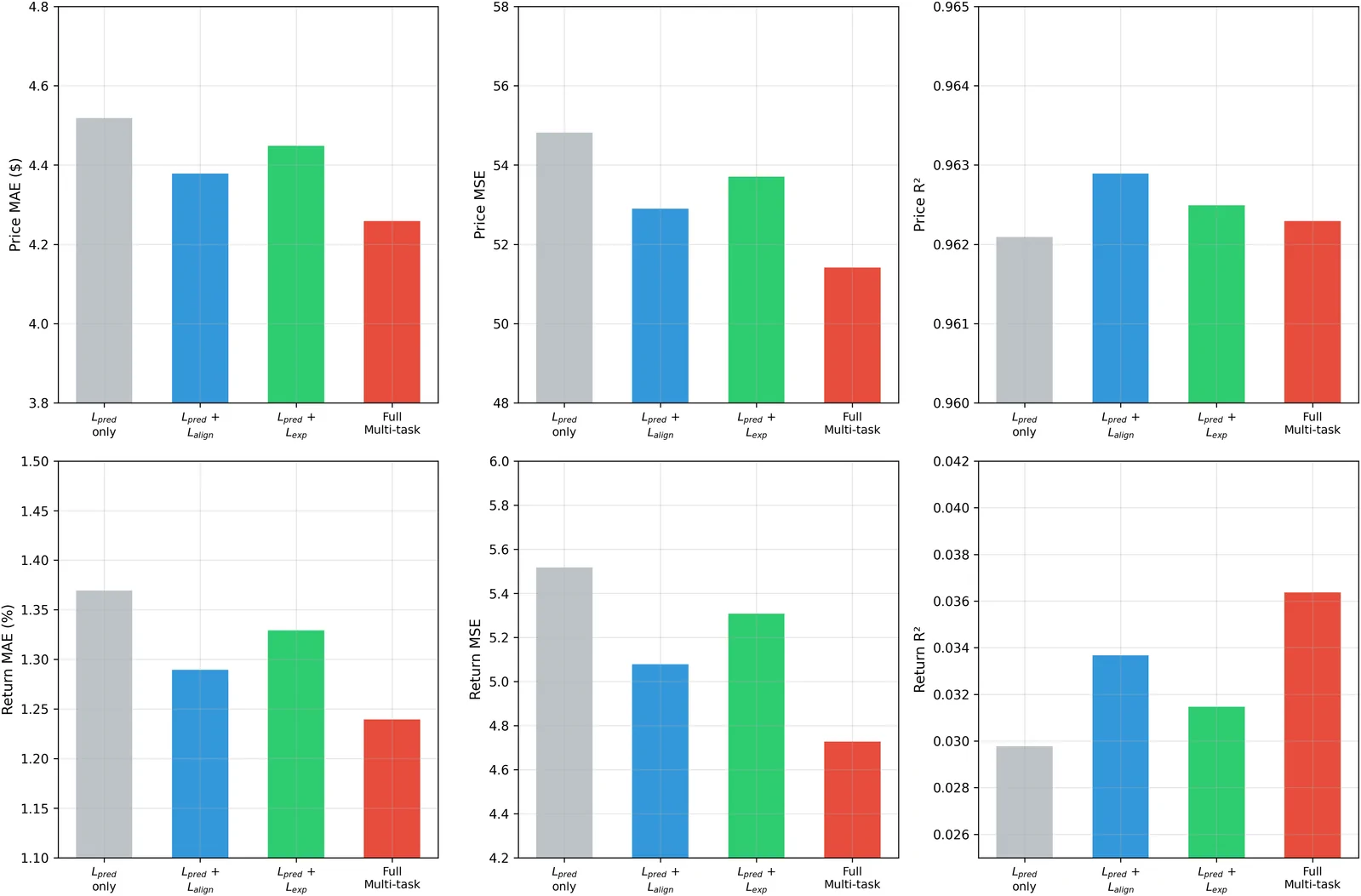
Loss function ablation using full STFA architecture. Progressive addition of alignment and explanation losses improves both prediction accuracy and model interpretability. All values are computed on the 50-stock test set (2022–2023).

Training with prediction loss only achieves return R^2^ = 0.0298, establishing the supervised learning baseline. This configuration optimizes solely for forecasting accuracy without explicit regularization on cross-modal representations or attention mechanisms.

Adding alignment loss improves return R^2^ to 0.0337, demonstrating the benefit of encouraging semantic and numerical representations to be mutually consistent. The alignment loss acts as a regularizer that prevents the two modalities from learning contradictory patterns. When news sentiment is strongly positive, the alignment loss encourages the model to also recognize upward price momentum, creating coherent multimodal representations.

Adding explanation loss instead yields return R^2^ = 0.0315, providing smaller but meaningful gains. This loss guides attention weights toward interpretable patterns by encouraging focus on event-relevant time steps. While primarily improving interpretability, it also acts as attention regularization that prevents overfitting to spurious patterns.

The complete multi-task loss combining all three terms achieves the best performance at return R^2^ = 0.0364 and price MAE = $4.26, matching the full STFA result in Table 2. The synergy between alignment and explanation losses is evident: alignment ensures cross-modal consistency while explanation ensures attention focuses on meaningful patterns. Together, they guide the model toward learning robust, interpretable representations that generalize better than prediction loss alone.

Comparing relative contributions, alignment loss provides larger gains than explanation loss (return R^2^ improvement of 0.0039 versus 0.0017), suggesting that cross-modal consistency is more critical than attention regularization for this task. However, the complete loss outperforms either individual auxiliary loss, demonstrating complementary benefits. The alignment loss shapes what the model learns, while the explanation loss shapes how it attends.

### Visualization analysis

Fig 6 demonstrates how STFA performs semantic-temporal alignment in an actual market prediction task using a representative case from the FNSPID dataset. The figure shows the model’s behavior around a financial news event released on May 10, 2022 (orange dashed line), which was associated with a specific company in the dataset. Based on the semantic representation produced by the GPT-4-supervised fine-tuned FinBERT encoder, STFA predicts that the market reaction would occur with a two-day delay on May 12, 2022 (red dashed line). This delay value (δ=2) is learned by the delay-aware module during training rather than predefined, reflecting the model’s capacity to capture deferred effects from textual events. Importantly, the prediction at time *T* uses only information available up to *T*, ensuring no look-ahead bias in the forecasting process.

**Fig 6:**
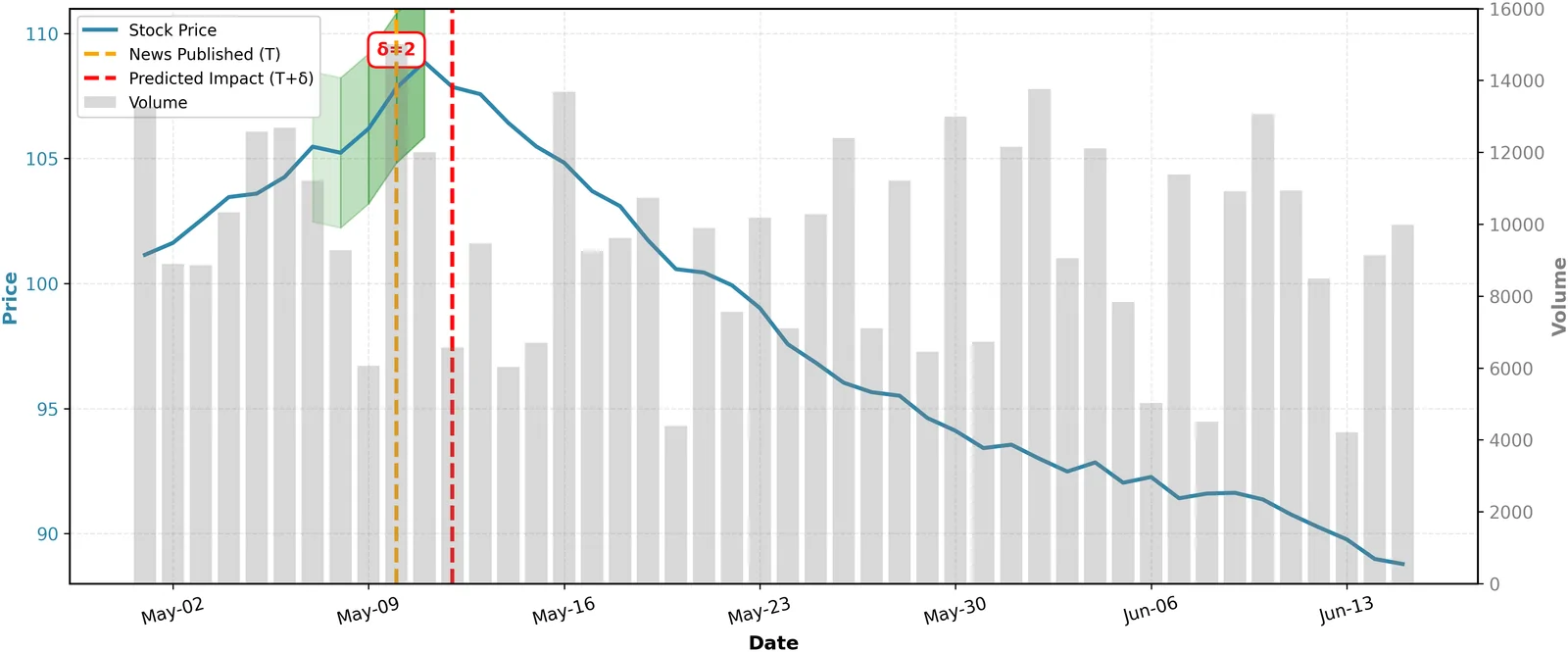
Temporal alignment of semantic and numerical signals in an STFA prediction case from the FNSPID dataset. The model predicts a two-day delay between news release and market reaction using only historical information.

The blue line shows the stock price trajectory over time, while gray bars represent daily trading volume. Green shaded bands overlaid on the price curve represent attention weights computed by the semantic-guided attention module, indicating which historical time steps the model deems most relevant when processing the news context. In this example, attention concentrates on the three trading days immediately preceding the news release (T−3 to *T*), highlighting STFA’s ability to identify semantically relevant market patterns prior to the event. The highest attention weight appears on the day of the news release, emphasizing the importance of the market state at the moment the semantic signal is introduced.

Fig 7 illustrates the convergence behavior of STFA over 100 training epochs, averaged across five independent runs with different random seeds. The figure shows three individual loss components (forecasting loss, semantic alignment loss, and interpretability loss) along with the weighted total loss used as the training objective ($L_{\text{total}}=1.0\cdot L_{\text{pred}}+0.5\cdot L_{\text{align}}+0.3\cdot L_{\text{exp}}$). Shaded regions indicate ±1 standard deviation across runs.

**Fig 7:**
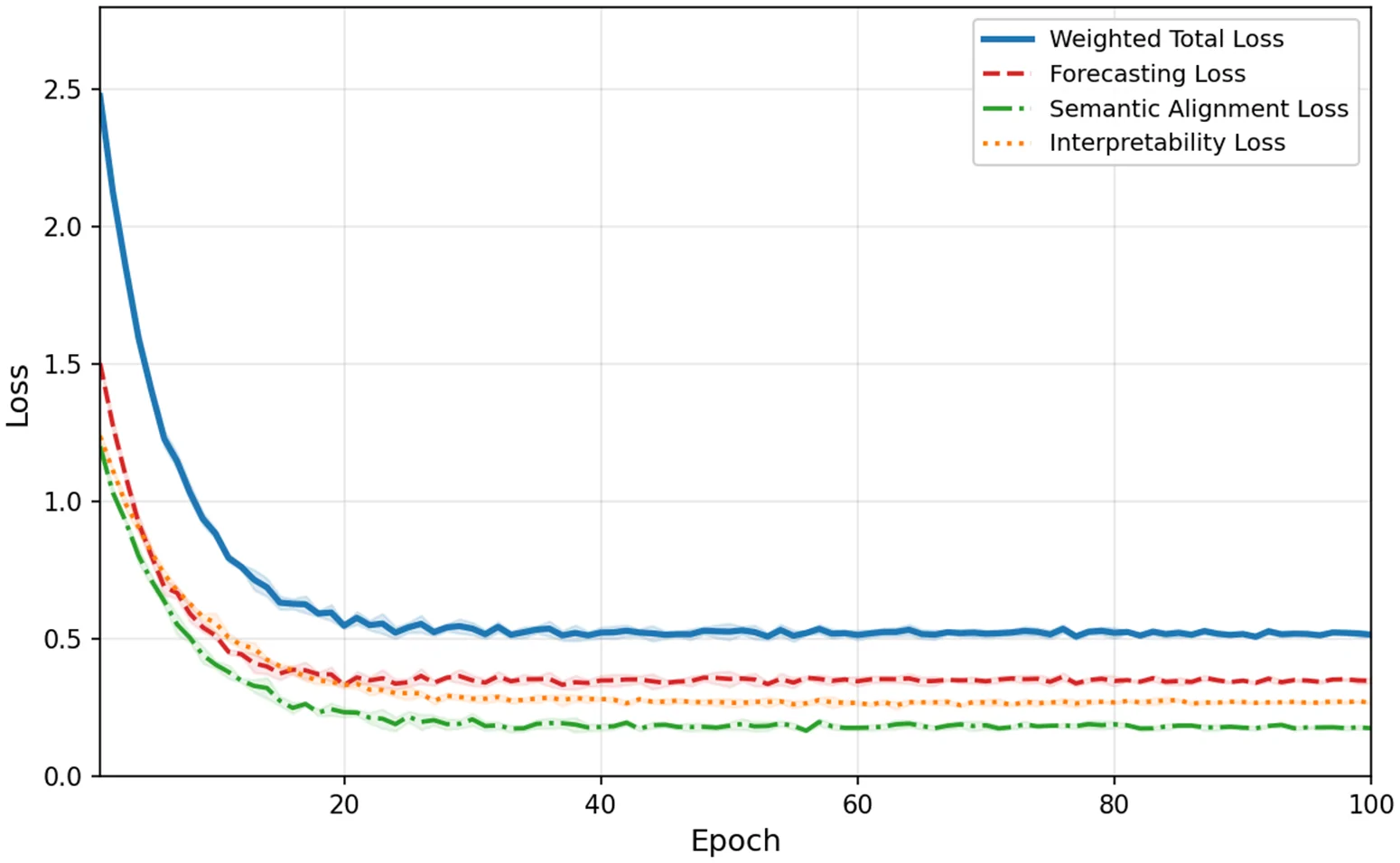
Training convergence of STFA, averaged over five independent runs. The weighted total loss ($1.0\cdot L_{\text{pred}}+0.5\cdot L_{\text{align}}+0.3\cdot L_{\text{exp}}$) and its three individual components are shown separately. Shaded regions denote ±1 standard deviation.

As training progresses, all three components exhibit monotonic downward trends. The forecasting loss decreases most rapidly, stabilizing around epoch 60, while the semantic alignment and interpretability losses converge more gradually, reflecting their dependence on higher-order textual and attention-based representations. The weighted total loss decreases from 2.47 at initialization to 0.52 by epoch 100, with narrow confidence bands throughout training indicating consistent optimization dynamics across runs. This convergence pattern confirms that the three objectives do not conflict during joint optimization and that STFA can simultaneously improve forecasting accuracy, semantic grounding, and interpretability within a single training procedure.

Fig 8 compares the semantic subspaces produced by four models: TextBlob, FinBERT, Loughran-McDonald, and the proposed STFA. For each model, semantic vectors corresponding to three categories (sentiment, event type, and temporal delay) are extracted from financial news inputs sampled from the test set. These high-dimensional representations are projected into two-dimensional space using Principal Component Analysis (PCA) to reveal latent structure.

**Fig 8:**
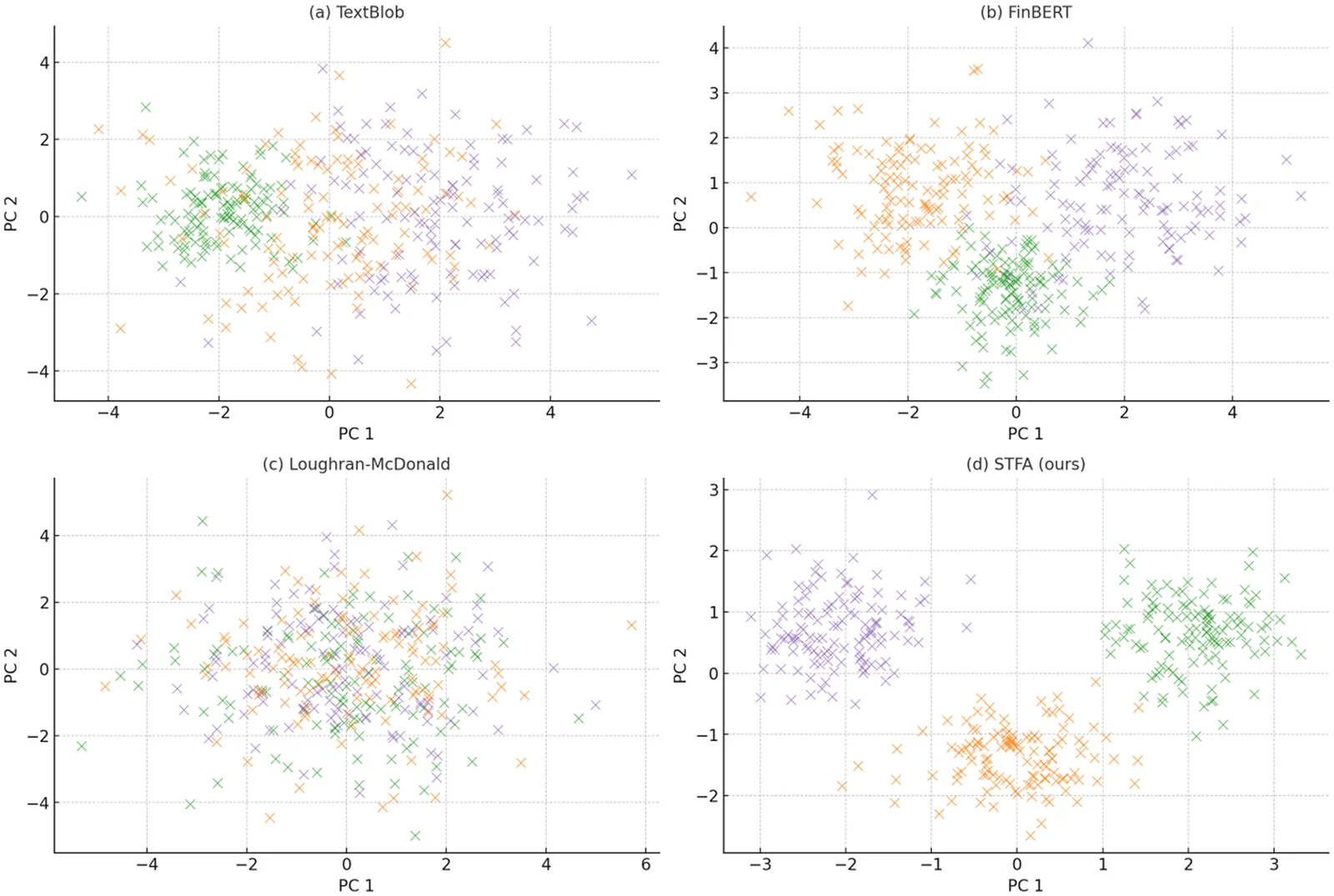
Cross-model comparison of semantic subspace representations visualized through PCA projection.

Subplot (a) shows that the lexicon-based TextBlob model produces overlapping and poorly separated semantic vectors, with no clear distinction between sentiment and non-sentiment dimensions. In (b), FinBERT, although pretrained on financial text, shows only modest improvement: sentiment vectors are slightly more compact, but event and delay representations remain entangled. The Loughran-McDonald lexicon in (c) performs similarly to TextBlob, reflecting its handcrafted nature and limited expressive capacity.

In contrast, STFA in (d) exhibits more structured separation across all three semantic types, with distinct clustering regions for sentiment, event type, and temporal delay. The improved separation demonstrates that STFA’s multi-headed semantic encoder, trained jointly with delay modeling and attention supervision, captures more disentangled and task-relevant representations. These structured semantic features support improved multimodal alignment and downstream forecasting performance.

Fig 9 illustrates how STFA dynamically allocates attention across historical time steps based on the semantics of incoming financial news. The heatmap displays attention weights for six representative semantic event types, including “Earnings Miss,” “Merger News,” “Guidance Cut,” “Rate Hike,” “CEO Resigns,” and “SEC Investigation.” Each row corresponds to one event type, with attention weights averaged over all occurrences of that category in the test set. Each column represents a day in the 20-day historical window prior to the news release. The brightness of each cell reflects the magnitude of attention assigned to that time step.

**Fig 9:**
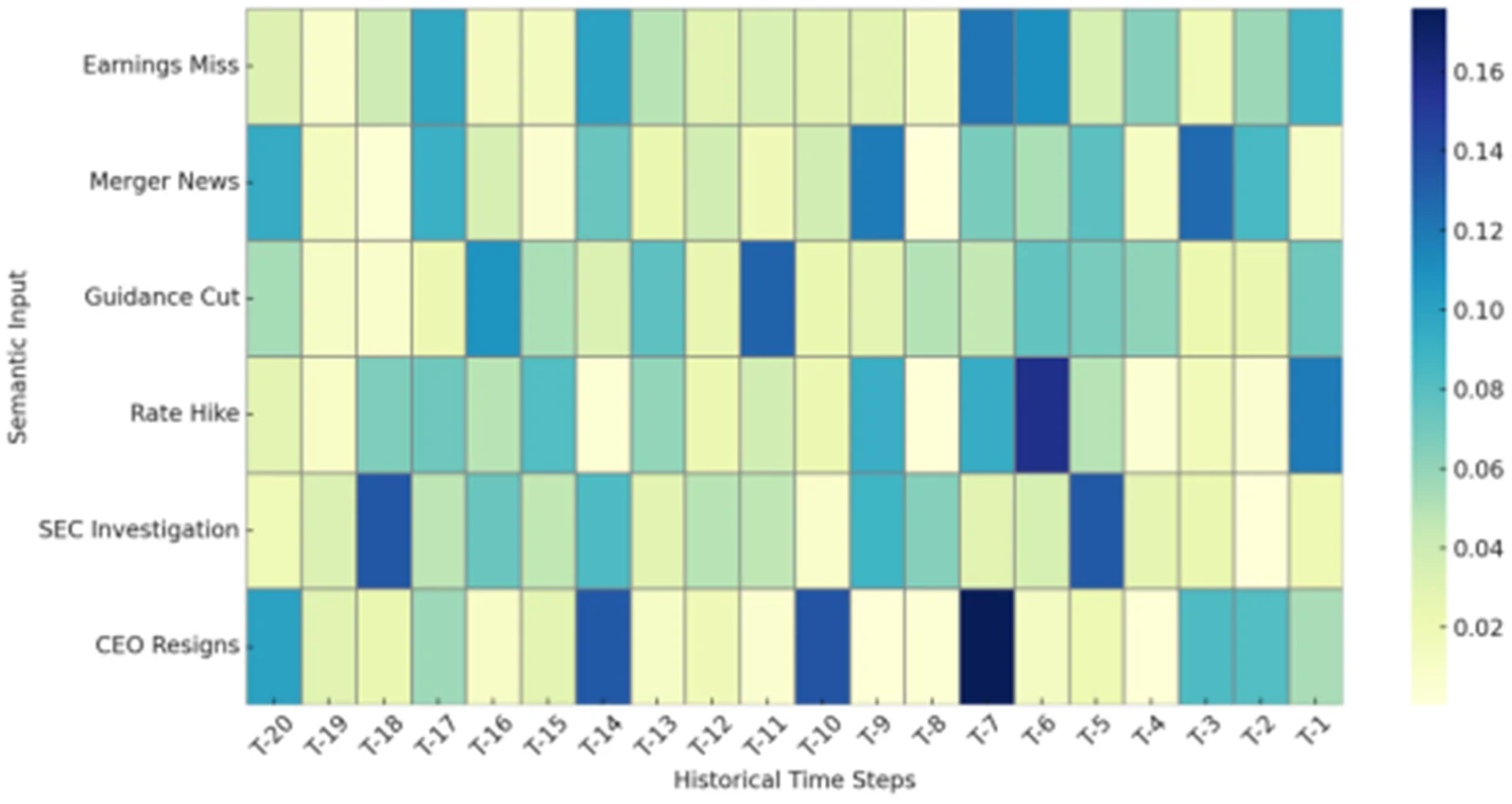
Semantic-guided temporal attention heatmap showing differential attention patterns across event types.

Forward-looking events such as “Guidance Cut” and “Rate Hike” concentrate attention on the most recent days (T−1 to T−3), indicating that the model relies on short-term technical context when reasoning about policy shifts or market signals with immediate implications. In contrast, events such as “SEC Investigation” and “CEO Resigns” display more distributed attention patterns, suggesting that the model integrates broader historical behavior when interpreting uncertain or delayed-impact governance events. This semantic-dependent attention allocation demonstrates STFA’s capacity to adapt temporal reasoning patterns based on news content, rather than applying uniform historical weighting across all event types.

## Conclusion

This work presents the Semantic-Time Fusion Architecture (STFA), a unified framework that integrates structured financial semantics with quantitative time-series data for enhanced market forecasting. Key contributions include a semantic encoding module that extracts sentiment, event cues, and temporal deferral information from unstructured financial text using GPT-4-supervised fine-tuned FinBERT representations, a delay-aware semantic alignment mechanism that models lagged effects between textual signals and market behavior, a multi-scale temporal fusion module that captures market reactions across different time horizons, and a joint training objective that balances prediction accuracy with semantic consistency and interpretability.

Extensive experiments on the FNSPID dataset demonstrate that STFA outperforms classical and deep learning baselines across multiple metrics. The model shows practical value through improved directional accuracy and superior Sharpe ratio performance compared with random walk, ARIMA, and Transformer baselines. Ablation studies confirm that removing semantic, delay-aware, attention-based, or multi-scale components reduces performance, while comparisons between GPT-4-supervised FinBERT semantic representations and TextBlob sentiment features highlight the importance of high-quality contextual semantic information. Statistical validation with confidence intervals, independent runs, and corrected significance tests supports the robustness of the reported improvements.

### Limitations

Several limitations of this work should be acknowledged. First, the model’s performance depends on the availability and quality of financial news coverage, which varies substantially across companies. Our stock sample was selected from S&P 500 firms with consistent news flow over the evaluation period, limiting generalizability to smaller entities or less-covered markets. The selection procedure prioritized data completeness over randomness, introducing potential sample selection bias toward larger, more newsworthy companies. Most notably, by requiring continuous listing from 2013 to 2023, the dataset embeds survivorship bias. Approximately 74% of the initial universe was filtered out by the combined eligibility criteria. As a result, the evaluation does not fully capture the model’s performance on distressed companies that delisted or went bankrupt during the sample period. While this ensures stable temporal windows for model training, it may upwardly bias the baseline market returns and alter volatility profiles compared to a point-in-time universe.

Second, the observed performance metrics should be interpreted within the context of percentage return prediction rather than absolute price forecasting. Financial time series exhibit complex nonlinear dynamics and regime shifts that are inherently difficult to capture with static models. Our evaluation period spanned a moderate-volatility regime relative to historical crisis periods, which may favor certain modeling approaches. Out-of-sample testing on more volatile periods or longer forecasting horizons would provide a more robust assessment of generalization.

Third, the current architecture assumes semantic signals remain stable within the prediction window, which may not hold during periods of rapid information arrival or market stress. The fixed-window attention mechanism does not adaptively adjust to varying information density across different market conditions. Extending the model to handle dynamic window sizes or intra-day news updates remains an important direction for future work.

Fourth, the GPT-4 annotation stage introduces computational and financial costs that may limit accessibility for smaller research teams or real-time deployment scenarios. Although the annotation pipeline and semantic feature generation procedure are documented to facilitate reproducibility, replicating the annotation process from scratch requires substantial resources. Alternative approaches using open-source language models, model distillation, or shared annotation protocols could help reduce this limitation.

Fifth, while our evaluation includes realistic transaction cost estimates (commission, bid-ask spread, and market impact averaging 15 basis points one-way as detailed in the Economic Evaluation Protocol), we do not account for additional practical constraints such as limited short-selling availability, margin requirements, portfolio rebalancing frictions at scale, or the full impact of large-scale order execution on market microstructure that would affect institutional-level deployment. Furthermore, our trading simulation assumes sufficient liquidity for all positions, which may not hold for less liquid stocks or during periods of market stress.

### Future directions

Future work includes several promising directions. First, developing dynamic sentiment models that adapt to intra-day news fluctuations and varying information density across market conditions. Second, extending the architecture to multimodal inputs such as earnings call audio, financial document images, and social media signals to capture richer contextual information. Third, improving interpretability through attention visualization, counterfactual analysis, and causal reasoning to support explainable decision-making. Fourth, integrating reinforcement learning for decision-aware forecasting tailored to specific trading strategies, risk management, and audit alert systems. Fifth, conducting large-scale robustness testing across different market regimes, asset classes, and international markets to assess model generalizability beyond the current sample.
